# Comparative Genomic Analysis Reveals Key Changes in the Genome of *Acremonium chrysogenum* That Occurred During Classical Strain Improvement for Production of Antibiotic Cephalosporin C

**DOI:** 10.3390/ijms26010181

**Published:** 2024-12-28

**Authors:** Alexander A. Zhgun

**Affiliations:** Group of Fungal Genetic Engineering, Federal Research Center “Fundamentals of Biotechnology” of the Russian Academy of Sciences, Leninsky Prosp. 33-2, 119071 Moscow, Russia; jgoun@biengi.ac.ru; Tel.: +7-916-9749769

**Keywords:** whole-genome sequencing, *Hapsidospora chrysogena*, cephalosporin C, antibiotics, secondary metabolites, mold fungi, classical strain improvement (CSI), high-yielding (HY) strain, biosynthetic gene cluster (BGC)

## Abstract

From the 1950s to the present, the main tool for obtaining fungal industrial producers of secondary metabolites remains the so-called classical strain improvement (CSI) methods associated with multi-round random mutagenesis and screening for the level of target products. As a result of the application of such techniques, the yield of target secondary metabolites in high-yielding (HY) strains was increased hundreds of times compared to the wild-type (WT) parental strains. However, the events that occur at the molecular level during CSI programs are still unknown. In this paper, an attempt was made to identify characteristic changes at the genome level that occurred during CSI of the *Acremonium chrysogenum* WT strain (ATCC 11550) and led to the creation of the *A. chrysogenum* HY strain (RNCM F-4081D), which produces 200–300 times more cephalosporin C, the starting substance for obtaining cephalosporin antibiotics of the 1st–5th generations. We identified 3730 mutational changes, 56 of which led to significant disturbances in protein synthesis and concern: (i) enzymes of primary and secondary metabolism; (ii) transporters, including MDR; (iii) regulators, including cell cycle and chromatin remodeling; (iv) other processes. There was also a focus on mutations occurring in the biosynthetic gene clusters (BGCs) of the HY strain; polyketide synthases were found to be hot spots for mutagenesis. The obtained data open up the possibility not only for understanding the molecular basis for the increase in cephalosporin C production in *A. chrysogenum* HY, but also show the universal events that occur when improving mold strains for the production of secondary metabolites by classical methods.

## 1. Introduction

*Acremonium chrysogenum* (now reclassified as *Hapsidospora chrysogena*) is the exclusive organism used for the industrial production of cephalosporin C (CPC), the source substance for the production of antibiotics of the cephalosporin class of 1–5 generations [1,2,3]. Cephalosporins are widely used in the pharmaceutical industry and account for more than 20 percent of the antibiotic market [4,5,6]. There are currently 24 cephalosporin antibiotics available for clinical use in the United States [7]. *A. chrysogenum* belongs to the class *Sordariomycetes*, and division *Ascomycota*. Currently, CPC biosynthesis has also been detected in a number of other fungi from the classes *Sordariomycetes* (*Kallichroma tethys*, *Pochonia chlamydosporia*) and *Eurotiomycetes* (*Paecilomyces persicinus*) [8,9,10,11]. However, only *A. chrysogenum* is used for industrial production of CPC [12]. This is due to extensive research into this organism over the last 70 years and the creation of highly active CPC producers through so-called classical strain improvement (CSI) programs [1,13,14]. The research conducted was reflected in a significant volume of publications, in which this organism was initially called *Cephalosporium acremonium*, then reclassified as *Acremonium chrysogenum*, and in most scientific publications this organism was listed under this name [3,15]. However, in 2023, *Acremonium chrysogenum* was reclassified again, this time to *Hapsidospora chrysogena* [16]. Since in our previous works, as well as in the works of other scientific teams with this organism, the name *A. chrysogenum* was used, in this study we continue to use exactly this name so as not to cause confusion, as other authors are doing now [17,18,19]. Similarly, after the reclassification of *Penicillium chrysogenum* strains (used for PenG production, both the original Fleming strain and the strains used to obtain improved industrial producers) as *Penicillium rubens* [20], authors of publications continue to use the name *P. chrysogenum* to avoid confusion [21].

This work is devoted to the study of changes that occurred in the genome of the *A. chrysogenum* high-yielding (HY) strain RNCM 408D, obtained on the basis of the *A. chrysogenum* wild-type (WT) strain ATCC 11550 as a result of the CSI program [22]. *A. chrysogenum* ATCC 11550 was isolated in 1945 by the Italian pharmacologist Giuseppe Brotzu from seawater near a sewage discharge in Cagliari (Sardinia, Italy) [23,24]. To obtain *A. chrysogenum* HY, multi-round random mutagenesis was used (a total of 47 mutagenesis and screening stages were performed) using UV irradiation and chemical mutagenesis with N-Nitroso-N-methylurea, N-Nitrosomethylbiuret, and 1-Methyl-3-nitro-1-nitrosoguanidined. At the first stages of mutagenesis, screening was performed for activity against *Alcaligenes faecalis* ATCC 8750, penicillin N-resistant strain, which allowed the selection of *A. chrysogenum* variants improved in the production of cephems (synthesized at the last stages of CPC biosynthesis) [10]. Then, screening was performed for the production of beta-lactams by HPLC, as described previously [25]. This made it possible to select strains with increased production of the target cephem, cephalosporin C, and to obtain the final strain HY with an insignificant amount of impurity intermediates of cephems, such as deacetoxycephalosporin C (DAOC) and deacetoxycephalosporin C (DAC) [26]. In parallel with the tests for CPC activity, undesirable auxotrophy was studied in the obtained mutants. At one of the stages of mutagenesis, the highly active producer became auxotrophic for methionine. Fermentation required the addition of L-methionine, accompanied by unpleasant odors from the resulting organosulfur compounds. However, subsequent rounds of mutagenesis allowed us to obtain a prototrophic revertant, the RNCM 408D strain does not require the addition of L-methionine for CPC biosynthesis, and its fermentation is not accompanied by the odors of organosulfur compounds. The CPC production level of this WT strain is 25–70 mg/L; for *A. chrysogenum* HY, the CPC yield is 9000–12,000 mg/L and more [27]. Along with this, other phenotypic changes occurred in *A. chrysogenum* HY during the CSI program, both at the morphological and biosynthetic levels (Table 1). On the one hand, they are associated with a general decrease in the viability of *A. chrysogenum* HY, expressed in a decrease in the size of colonies, loss of aerial mycelium, reduction in the formation of conidia, loss of yellow-cream coloration on complex agarized (CPA) medium [26,28] and thinning of the cell wall, a decrease in biomass, a decrease in intracellular polyamine content and decrease in the activity of plasma membrane H^+^-ATPase during submerged fermentation on complex (CP) medium [27,29,30,31]. Also on the CPA medium, colonies of *A. chrysogenum* HY, in contrast to the wild-type parental strain, are heterogeneous in size (along with a general decrease in size) [26]; during submerged fermentation in CP medium, starting from the middle of fermentation, the mycelium of *A. chrysogenum* HY is reduced to so-called arthrospores (oidia), actively producing CPC [32]. On the other hand, the HY strain exhibits significant and unexpected resistance to inhibitors of the key enzyme of polyamine biosynthesis, ornithine decarboxylase (ODC, EC: 4.1.1.17) on Czapek-Dox agarized (CDA) medium [33,34]. ODC inhibitors such as α-difluoromethylornithine (DFMO) and APA (1-aminooxy-3-aminopropane) at a concentration of 5 mM completely inhibited the growth of *A. chrysogenum* WT and had virtually no effect on the growth of *A. chrysogenum* HY [33]. At the same time, a significant, 4–5 times increase in the content of the polyamine pool, represented by spermidine and spermine, was observed in *A. chrysogenum* HY [33]. It was also shown that exogenous administration of spermidine and 1,3-diaminopropane (1,3-DAP) can have a significant effect on the biosynthesis of CPC, increasing its yield by 15% or more [26], or lead to the accumulation of its precursor (during fermentation on a selected medium with 1,3-DAP), while the total yield of cephems increases, and the production of CPC decreases [25].

In addition to the phenotypic differences between the *A. chrysogenum* WT and HY strains, which are associated with alterations in morphology and a number of biochemical differences, some changes at the molecular level were also detected. As a result of molecular karyotyping, it was shown that in *A. chrysogenum* HY, significant chromosomal rearrangements occurred, leading to changes in chromosome size, which, however, did not affect the loci and chromosomes where the so-called “early” and “late” biosynthetic gene clusters (BGCs) of beta-lactams (necessary for the biosynthesis of CPC) are clustered [35]. Also, no increase in gene dosage was detected for beta-lactam BGCs; for *A. chrysogenum* HY, independent methods showed the presence of one copy of “early” genes and one copy of “late” genes, as in the genome of the parental strain *A. chrysogenum* WT [27,35]. This is important data, since in high-yielding fungal producers improved by classical methods, increased production of a secondary metabolite may be accompanied by duplication of the target BGC [36,37], including multiple duplications [38,39,40,41,42]. At the same time, in *A. chrysogenum* HY for CPC biosynthesis genes, upregulation was 5–300 times throughout the entire fermentation period [27].

In this regard, the aim of this work was to conduct a comparative genomic analysis of *A. chrysogenum* WT and *A. chrysogenum* HY strains with a focus on attempting to explain the previously demonstrated changes that occurred at the levels of macro- and micromorphology, karyotype, gene expression, and metabolism as a result of the CSI program. Whole genome sequencing of *A. chrysogenum* HY and comparative analysis revealed a significant number of SNPs, 3472, many of which appear to be noise associated with side mutations. However, it is possible to identify several groups of changes associated with the increase in CPC production, such as (i) disruption in BGCs for the biosynthesis of alternative secondary metabolites, especially in polyketide synthases; (ii) no changes in target BGCs for CPC biosynthesis; (iii) mutations in primary metabolism enzymes associated with the redistribution of fluxes in favor of the primary metabolites of target metabolism; (iv) mutations in transporter proteins, including MDR; (v) changes in the regulatory system, including those associated with cell cycle control, transport of macromolecules between the nucleus and cytoplasm, and chromatin remodeling; (vi) other changes associated with the control of glycosylation, folding, sporulation, etc. A special focus is placed on the changes in the biosynthetic potential of *A. chrysogenum* HY in light of mutations occurring in BGCs.

## 2. Results

### 2.1. Mutations That Occurred in A. chrysogenum HY by Types and Categories

The genome of *A. chrysogenum* strain HY was sequenced using the Illumina NGS platform at 297×coverage. As a result of comparison of the genome sequences of *A. chrysogenum* strain HY with the original parental strain *A. chrysogenum* WT, 3472 SNPs were found, leading to 3730 mutational changes in the categories: (i) HIGH—56 changes; (ii) MODERATE—525 changes; (iii) LOW—373 changes; and (iv) MODIFIER—2769 changes (Table 2 and Table S1). The discrepancy between the number of SNPs in the genome and specific mutations by category is due to the fact that the same SNP can affect several genes and belong to different categories. For example, the MODERATE missense mutation c.3019G>T in ACRE_001320 protein (resulting from nucleotide substitution p.Gly1007Trp in the KFH48836 transcript) is also defined as a MODIFIER mutation for ACRE_001330 (c.*909C>A downstream gene variant).

**Figure 1:**
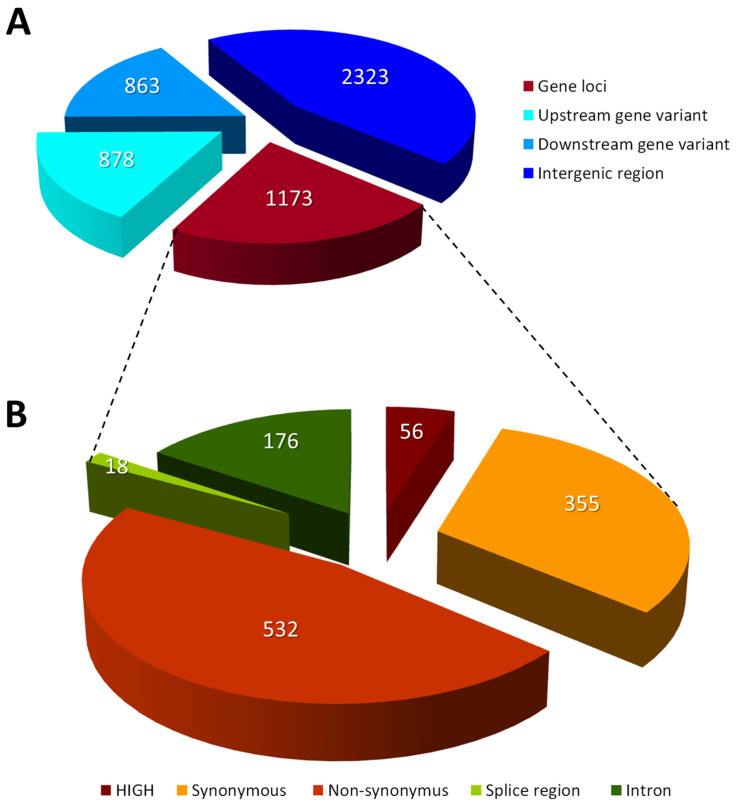
Mutations that occurred in *A. chrysogenum* HY during the classical strain improvement program. (**A**)—Distribution of mutations between gene loci, upstream/downstream regulatory regions, and intergenic regions. (**B**)—Distribution of mutations affecting the gene loci. Category HIGH includes the following mutations: frameshift—23 mutations, stop gained—21, stop lost—9, splice acceptor—2, and splice donor—1.

The most significant changes (leading to disruption of protein production) from the HIGH category are (i) frameshift variant—22 cases; (ii) frameshift variant and start lost—1 case; (iii) stop gained—19 cases; (iv) stop gained and splice region variant—2 cases; (v) stop lost—9 cases; (vi) splice acceptor variant and intron variant—2 cases; (vii) splice donor variant and intron variant—1 case (Figure 1, Tables S1 and S2). In the MODERATE category, the majority of SNPs are associated with a missense variant (527 cases); there are also 2 cases with conservative inframe deletion and 1 case each with disruptive inframe insertion, conservative inframe insertion, and disruptive inframe deletion (Table S2). Mutations from the LOW category are represented mostly by synonymous variant (in 355 cases); in addition, splice region variant and synonymous variant (in 4 cases) and splice region variant and intron variant (in 14 cases) are found. Although synonymous mutations generally coincide to have minor effects, emerging evidence suggests that such mutations can influence fitness through a variety of molecular mechanisms, including the creation of illicit RNA polymerase binding sites that affect transcription and changes in mRNA folding stability that modulate translation [43].

The largest number of mutations that have arisen belong to the MODIFIER category and are located in intergenic regions (2323 cases); upstream (878) or downstream (863) from genes; or in introns (176). Since the same mutation belonging to the MODIFIER category can have effects on different genes, for example, occupy an upstream position for one gene and occupy a downstream position for another gene, the total number of these effects, 4240 (in intergenic regions, upstream/downstream from genes or in introns) significantly exceeds the number of modification-type mutations in the genome, 2769 (Table 2 and Table S1).

As a result of comparative analysis in the *A. chrysogenum* HY genome, a significant number of mutational changes were identified, including those related to the HIGH and MODERATE categories. However, the question arises as to which changes contribute to the production of highly active CPC products, and which are related to noise and are side mutations resulting from random mutagenesis.

### 2.2. Analysis of the Metabolic Repertoire of A. chrysogenum Strains

#### 2.2.1. Secondary Metabolites Produced by *A. chrysogenum* WT Strain

*A. chrysogenum* ATCC 11550 is known to produce a variety of secondary metabolites. On the ATCC collection page, it is stated that this strain produces cephalosporin C, cephalosporin P, deacetoxycephalosporin C, penicillin N, adicillin, cephalosporin N, isopenicillin N, and synnematin B [24]. Most of these compounds are products of the CPC biosynthetic pathway and are obtained as a result of the activity of biosynthetic enzymes from “early” and “late” BGCs of beta-lactams. The pathway of transformation of these secondary metabolites is isopenicillin N → penicillin N (also called adicillin, or cephalosporin N, or synnematin B [44]) → deacetoxycephalosporin C → cephalosporin C (CPC).

The cephalosporin P fraction contains five antibiotics, which have been named cephalosporin P_1_, P_2_, P_3_, P_4_, and P_5_ [45]. The major component of cephalosporin P fraction is cephalosporin P_1_. Its biosynthesis occurs as a result of the functioning of two BGCs, one of which contains six biosynthetic genes: *cepA* (for oxidosqualene cyclase), *cepB1* (cytochrome P450), *cepB2* (cytochrome P450), *cepB3* (cytochrome P450), *cepC1* (the short-chain dehydrogenase/reductase), and *cepD1* (acyltransferase) [46]. The second BGC contains three genes for tailoring enzymes: *cepC2* (for short-chain dehydrogenase/reductase), *cepB4* (cytochrome P450), and *cepD2* (acyltransferase).

A series of recent studies have shown that sorbicillinoids, hexaketide secondary metabolites, are responsible for the characteristic yellow-cream coloration of *A. chrysogenum* WT [47,48]. The sorbicillinoid BGC contains two backbone PKSs (*sorA* and *sorB*), three tailor biosynthetic enzymes (*sorC*, *sorD*, and *sorE*), a transporter (*sorT*), and two pathway-specific regulators (*sorR1* and *sorR2*) [49]. As a result of the functioning of this BGC, *A. chrysogenum* WT is capable of producing at least 12 pigment compounds of this class. At the first step, sorbicillin and dihydrosorbicillin are synthesized as a result of the activity of SorA and SorB, and then they are oxidatively dearomatized by SorC to form sorbicillinol and dihydrosorbicillinol. These compounds are used as substrates in branched chemical reactions to produce five sorbicillinoids previously isolated from other fungi (trichotetronine, trichodimerol, demethyltrichodimerol, trichopyrone, and oxosorbicillinol) and three compounds isolated for the first time from *A. chrysogenum,* acresorbicillinols A–C [49].

There are also studies on the determination of siderophores in *A. chrysogenum*, such as dimerumic acid, coprogen B, 2-N-methylcoprogen B, and dimethylcoprogen [50]. At present, no BGCs have been identified for the production of these siderophores. The authors suggest that coprogen B, 2-N-methylcoprogen B, and dimethylcoprogen may be products of the same biosynthetic pathway and that dimerumic acid may be a degradation product of the other three coprogen-type siderophores.

#### 2.2.2. In Silico Analysis of BGC in *A. chrysogenum* WT Strain

For model fungi such as *Aspergillus nidulans* or *Penicillium chrysogenum*, both BGCs for known secondary metabolites and in silico calculated “orphan” BGCs are mapped to the corresponding chromosomal loci [51,52]. However, for *A. chrysogenum,* there is currently no genome-wide assembly with scaffolds assembled down to individual chromosomes. Deposited sequence for the genome of *A. chrysogenum* ATCC 11550 assembled into 541 scaffolds [53]. In this regard, chromosomal localization is known only for the best-characterized BGCs, such as the “early” and “late” biosynthetic clusters of beta-lactams, for which localization was established, not bioinformatically, but as a result of experiments, for example, after hybridization of probes (amplified fragments from these BGCs with an introduced label) onto chromosomes separated after pulsed-field gel electrophoresis [35].

Following the determination of the genome sequence of *A chrysogenum* WT in 2014, 42 BGCs were found: 7 NRPS (nonribosomal peptide synthetase), 14 PKS (polyketide synthetase), 10 TPC (terpene cyclase), 8 hybrid clusters, and 3 other BGCs (Table S3) [53]. To search for BGCs, D. Terfehr, et al. used the program antiSMASH v. 2.0. Currently, the antiSMASH v. 7.0 version is available online, which, according to the developers of the program, has an improved BGC search algorithm [54]. We used it to study the annotated genome sequence of *A. chrysogenum* ATCC 11550 (GenBank accession no. JPKY00000000.1) and found 50 BGCs: 9 NRPS, 14 PKS, 9 TPC, 9 hybrid clusters, and 9 other BGCs (Table S3). In particular, three additional NRPS were found, one of which was found near a TPC. Therefore, the number of hybrid clusters increased by one, the number of TPCs decreased by one, and the total number of NRPS increased by two (the third is located in a hybrid cluster). Also found were six clusters that fall into the “other” BGCs category. One of these is the BGC for isocyanide biosynthesis, which core enzymes, isocyanide synthase (ICS), are non-canonical and have not previously been included in genome analysis software [55].

In total, the biosynthetic repertoire of *A. chrysogenum* ATCC 11550 is represented by 53 backbone enzymes: (i) 14 NRPS, 9 of which (NRPS 1–9) are the only backbone enzymes of BGC, and 5 (NRPS 10–14) are part of hybrid clusters; (ii) 21 PKS, 15 of which are in 14 PKS clusters (PKS 1–3, PKS 4.1, PKS 4.2, PKS 5–14), and 6 (PKS 15–20) are part of hybrid clusters; (iii) 3 PKS/NRPS hybrid enzymes; (iv) 11 TPCs, 9 of which (TPC 1–9) are in individual clusters and 2 (TPC 10–11) are in hybrid clusters; (v) 3 core enzymes for indole biosynthesis, 1 in an individual cluster (IND 1) and 2 in hybrid clusters (IND 2–3); (vi) 1 cluster for isocyanide biosynthesis.

In this work, backbone enzyme numbers are entered in order of occurrence in the annotated sequence of the reference strain *A. chrysogenum* WT, GenBank accession number: JPKY00000000.1, [53]. Moreover, first, the backbone enzymes found in individual clusters are numbered in order, and then the numbering for them continues in the order of their occurrence in hybrid clusters. For example, *A. chrysogenum* WT has 14 PKS clusters. The PKS from the first individual cluster (encoded by the sequence ACRE_024990) was named PKS 1, the PKS from the second cluster (encoded by the sequence ACRE_034530, which has a later sequence number than PKS 1) was named PKS 2, etc. To maintain the correspondence between the number of PKS clusters and the PKS number, for the sorbicillinoid cluster (PKS 4), where two PKS function, they were named PKS 4.1 and PKS 4.2. After the last PKS assembled in the individual cluster, PKS14 (ACRE_088080), the numbering of PKSs assembled in hybrid BGCs continues: PKS 15 (ACRE_016830) from the 1st hybrid BGC, PKS 16 (ACRE_038250) from the 2nd hybrid BGC, etc.

Although several dozen secondary metabolites have been described for *A. chrysogenum*, most of these compounds are produced by the functioning of only a few BGCs. Most of the BGCs in this organism are currently “orphans”, i.e., their final low-molecular product(s) have not been identified. This study did not aim to mine BGCs and find products for “orphan” clusters. However, we have shown that many alternative BGCs, especially PKS, were significantly affected by the CSI program in *A. chrysogenum* HY, while the cephalosporin C targeting “early” and “late” beta-lactam BGCs were not affected.

### 2.3. Mutations in BGCs of A. chrysogenum HY

Since CSI programs for fungal strains have been shown to significantly affect alternative BGCs [41], we performed a comparative analysis of the gene clusters of *A. chrysogenum* WT and HY and characterized the detected mutational changes.

#### 2.3.1. Mutations in BGCs with NRPS in *A. chrysogenum* HY

**Figure 2:**
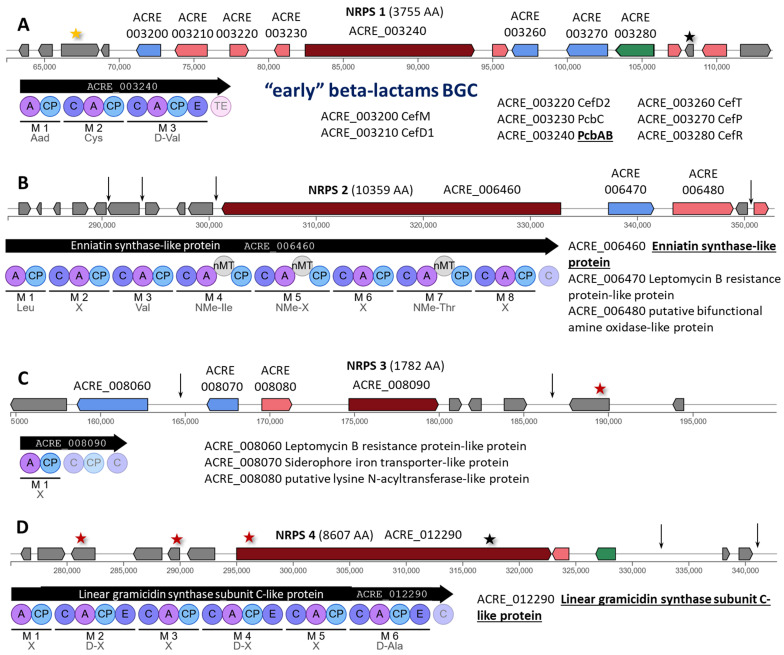
Mutational impact of the CSI program on secondary metabolite gene clusters with NRPS 1–4 in *A. chrysogenum* HY: (**A**)—NRPS 1, (**B**)—NRPS 2, (**C**)—NRPS 3, and (**D**)—NRPS 4. Sequences for NRPS are filled in brown boxes, biosynthetic tailoring enzymes are filled in red boxes, transporters are filled in blue boxes, path-specific regulators are filled in green boxes, and genes with unknown functions for secondary metabolite production are filled in gray boxes. Mutations in exons are indicated by asterisks filled in colors according to the following categories: HIGH—black, MODERATE—red, and LOW—yellow. Arrows indicate sites for SNPs outside the exons. Domains within NRPS modules are indicated by circles with the following inscribed letters: A—adenylation; CP—peptidyl carrier protein (PCP); C—condensation; E—epimerase; TE—thioesterase; and nMT—nitrogen methyltransferase.

**Figure 3:**
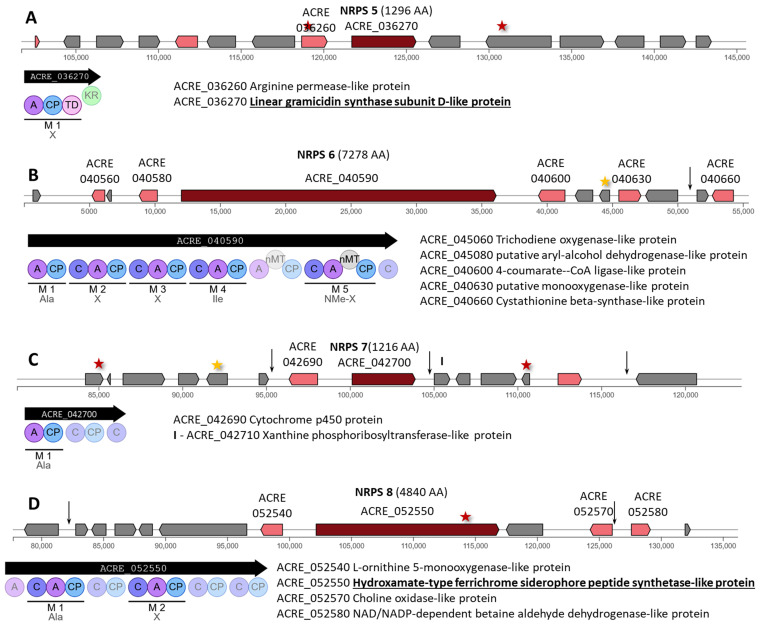
Mutational impact of the CSI program on secondary metabolite gene clusters with NRPS 5–8 in *A. chrysogenum* HY: (**A**)—NRPS 5, (**B**)—NRPS 6, (**C**)—NRPS 7, and (**D**)—NRPS 8. Sequences for NRPS are filled in brown boxes, biosynthetic tailoring enzymes are filled in red boxes, and genes with unknown functions for secondary metabolite production are filled in gray boxes. Mutations in exons are indicated by asterisks filled in colors according to the following categories: MODERATE—red, LOW—yellow. Arrows indicate sites for SNPs outside the exons. Domains within NRPS modules are indicated by circles with the following inscribed letters: A—adenylation; CP—peptidyl carrier protein (PCP); C—condensation; TD—terminal reductase; KR—ketoreductase; and nMT—nitrogen methyltransferase.

In *A. chrysogenum*, 9 of 14 NRPS are in individual clusters. Among them, mutations were detected for the 7 clusters in the HY strain, NRPS 2–8 (Figure 2 and Figure 3). For NRPS 1, two mutations were detected, located at the left and right boundaries of the cluster; for NRPS 9, no mutations were detected either within the BGC or near its boundaries.

The first NRPS (in order of occurrence in the annotated sequence of the reference strain *A. chrysogenum* WT, GenBank accession number: JPKY00000000.1, [53]) contains the “early” BGC of beta-lactams. The functioning of this cluster in *A. chrysogenum* has been studied in detail. NRPS 1 contains 3755 amino acid residues (AA) and is encoded by the gene ACRE_003240. This trimodular megasynthase, also known as PcbAB or ACV (δ-[L-α-Aminoadipyl]-L-Cysteinyl-D-Valine) synthetase (EC: 6.3.2.26), polymerizes the LLD-ACV tripeptide—δ-(L-α-aminoadipoyl)-L-cysteinyl-D-valine. Then, PcbC (encoded by ACRE_003230), isopenicillin N-synthase (EC: 1.21.3.1), as a result of a dioxygenase reaction, cyclizes this tripeptide to isopenicillin N (IPN); then cefD1 (encoded by ACRE_003220), isopenicillin N-CoA synthetase (EC: 5.1.1.17), and cefD2 (encoded by ACRE_003210), isopenicillin N-CoA epimerase (EC: 5.1.1.17), catalyze reactions leading to IPN epimyrization to penicillin N (penN); followed by enzymes of the “late” beta-lactam BGC, CefEF (encoded by ACRE_042800), deacetoxycephalosporin C synthetase (penicillin N expandase, EC: 1.14.20.1)/deacetoxycephalosporin C hydroxylase (EC: 1.14.11.26) catalyze two successive reactions, converting penN to DAOC, then converting DAOC to DAC and CefG (encoded by ACRE_042790), deacetylcephalosporin-C acetyltransferase (EC: 2.3. 1.175), converting DAC to CPC [31,56,57]. This cluster also contains three transporter genes encoding (i) CefP (encoded by ACRE_003270) for the transfer of IPN from the cytoplasm to the peroxisome [58], where IPN epimerization to PenG occurs [56]; (ii) CefM (encoded by ACRE_003200) for the transfer of PenG from the peroxisome to the cytoplasm [59]; and (iii) CefT (encoded by ACRE_003260) for the transport of intermediates of the CPC biosynthesis pathway (IPN, PenG, DAOC, DAC) from the cell [60]. Also, the “early” BGC beta-lactam cluster contains a gene (ACRE_003280) for the CefR regulator, which is a pathway-specific negative regulator for the *cefT* gene and cross-cluster positive regulator for the *cefEF* from the “late” BGC [61].

In *A. chrysogenum* HY, no SNPs were found within the NRPS 1 (also known as “early” beta-lactams) cluster, and this is important data since the target production of the CPC depends on the functioning of this cluster. The absence of mutational changes both at the level of the coding sequences and in the regulatory regions of genes against the background of significant upregulation of this BGC [27] and in the absence of chromosomal rearrangements [35] may indicate the role of trans-acting factors, such as cross-cluster or global regulators [15]. In addition, mutations were found that were in close proximity to the boundaries of this cluster (Figure 2A, Table S4). On the left side of the cluster boundary in the ACRE_003180 transcript, a synonymous substitution c.474T>C|p.Pro158Pro occurred. On the right side of the ACRE_003300 transcript, a frameshift mutation c.332delG|p.Gly111fs occurred, which is also a downstream gene variant for ACRE_003290 (c.*328delC).

In the “late” BGC of beta-lactams, one mutation occurred near ACRE_042680, encoding *cefG*, a downstream gene variant c.*57C>G (Table S4). Perhaps this downstream mutation contributes to high *cefG* expression observed in *A. chrysogenum* HY [27], resulting in efficient conversion of DAC to CPC, with DAC admixture being 15% or less of the CPC content [26].

NRPS 2 (ACRE_006460) encodes an 8-module megasynthase of 10,359 AA with an unknown product (annotated as enniatin synthase-like protein), characterized by the presence of a nitrogen methyltransferase domain in modules 4, 5, and 7 (Figure 2B). A mutation in the regulatory region was found for this gene, downstream gene variant c.*92A>T (Table S4). Three more regulatory mutations were also found in intergenic regions: two in the region of the left border of this cluster and one in the region of the right border.

NRPS 3 (ACRE_008090) appears to encode a single-modular NRPS-dependent siderophore (of 1782 AA). This is possible because two transport proteins are assembled nearby, one of which, ACRE_008070, is annotated as a siderophore iron transporter-like protein (Figure 2C). In *A. chrysogenum* HY, this cluster has a mutation in the region between the transporter genes and also has one regulatory and one missense mutation on the right border of the cluster (Table S4).

NRPS 4 (annotated as linear gramicidin synthase subunit C-like protein) has been significantly damaged by mutagenesis (Figure 2D). Most likely, *A. chrysogenum* HY has lost the ability to synthesize both the final product and all its intermediates from this cluster. A stop gained mutation c.19633C>T|p.Gln6545* occurred in the gene (ACRE_012290) for the 8607 AA 6-modular NRPS, leading to the production of a truncated 6545 AA product instead of the full-length 8607 AA product. In addition, another mutation occurred at the beginning of this gene, resulting in a non-synonymous replacement of the negatively charged AA with a positive one, c.298G>A|p.Glu100Lys. Two more mutations leading to non-synonymous substitutions were found in the region of this cluster: 1093T>A|p.Phe365Ile in the ACRE_012240 gene and c.124G>A|p.Glu42Lys in the ACRE_012270 sequence. Also, in the region of the right border of the cluster, two mutations arose in the intergenic regions.

NRPS 5 (ACRE_036270) encodes a single-modular enzyme of 1296 AA, annotated as a linear gramicidin synthase subunit D-like protein (Figure 3A). Two missense mutations have arisen in this BGC: c.467C>T|p.Ala156Val in the ACRE_036260 gene for arginine permease-like protein and c.2426C>T|p.Ser809Phe in ACRE_036290.

NRPS 6 (ACRE_040590) encodes a five-module megasynthase of 7278 AA with a methyltransferase domain in module 5 (Figure 3B). Two mutations were found in this cluster, one of which led to a synonymous substitution c.42A>T|p.Ile14Ile in ACRE_040620 and is also an upstream gene variant c.-744T>A for the putative monooxygenase-like protein gene ACRE_040630. The second mutation occurred in the intergenic region ACRE_040640/ACRE_040650.

NRPS 7 (ACRE_042700) encodes a single-modular enzyme of 1216 AA (Figure 3C). In the downstream region of this transcript, a mutation n.104906T>C occurred. This cluster also contained mutations from the MODIFIER category in the downstream region of the ACRE_042690 gene for cytochrome P450 and in the intergenic region ACRE_042760-ACRE_042770. In addition, a missense mutation c.664C>T|p.Pro222Ser in ACRE_042630, a synonymous substitution c.852C>T|p.Gly284Gly in ACRE_042670, and a missense mutation c.70G>A|p.Glu24Lys in ACRE_042740 arose.

NRPS 8 (ACRE_052550, annotated as the gene for hydroxamate-type ferrichrome siderophore peptide synthetase-like protein) encodes a bimodular megasynthase of 4840 AA (Figure 3D). This 4840 AA megasynthase has undergone a p.Gln3605Lys substitution resulting in a c.10813C>A mutation (Table S4). Such a mutation can significantly affect the production of the target siderophore since it has been shown that missense mutations in megasynthases can lead to complete inhibition of the corresponding biosynthetic pathway [62]. Apparently, this cluster is used by *A. chrysogenum* WT for the synthesis of siderophore, since in addition to the gene for hydroxamate-type ferrichrome siderophore peptide synthetase-like protein, the gene for L-ornithine 5-monooxygenase-like protein is also assembled here. In the postulated NRPS-dependent pathway of siderophore biosynthesis in fungi, the initial biosynthetic step shared by pathways of both intra- and extracellular siderophore biosynthesis is associated with the conversion of L-ornithine to N^5^-hydroxy-L-ornithine by the enzyme ornithine-N^5^-monooxygenase, SidA (for *Aspergillus fumigatus*) [63]. This cluster also contained mutations c.-240T>C, upstream ACRE_052570 gene for choline oxidase-like protein. In addition, a mutation occurred in the intergenic region near the left border of the cluster.

NRPS 9 (ACRE_069520) encodes a single-modular enzyme of 1686 AA. No mutations were detected in this BGC or near its boundaries.

As a result, it can be concluded that among the nine clusters with NRPS, NRPS 4, for which the megasynthase is annotated as linear gramicidin synthase subunit C-like protein, suffered the most as a result of mutagenesis. The stop gained mutation p.Gln6545* in this protein not only prevents the synthesis of subunit C of linear gramicidin but also makes the work of other enzymes from other BGCs that form other subunits of linear gramicidin ineffective, since according to the canon, gramicidin is assembled by four multimodular nonribosomal peptide synthetases [64]. However, the function of NRPS 4 has not been clarified experimentally, and this megasynthase may function alone to construct the final metabolite and not be involved in gramicidin biosynthesis as predicted in silico. In NRPS 8, a nonsynonymous substitution p.Gln3605Lys occurred for siderophore synthesis, which could potentially significantly affect the synthesis of this compound. In the “early” beta-lactam cluster, NRPS 1, no mutations occurred, but two mutations were detected in genes of unknown function located near the left and right borders of the cluster. In clusters with NRPS 2, 3, 5–7, mutations were found in genes of tailoring enzymes, genes of unknown function, and in intergenic regions. In NRPS 9, no mutations were found.

#### 2.3.2. Mutations in BGCs with PKS in *A. chrysogenum* HY

**Figure 4:**
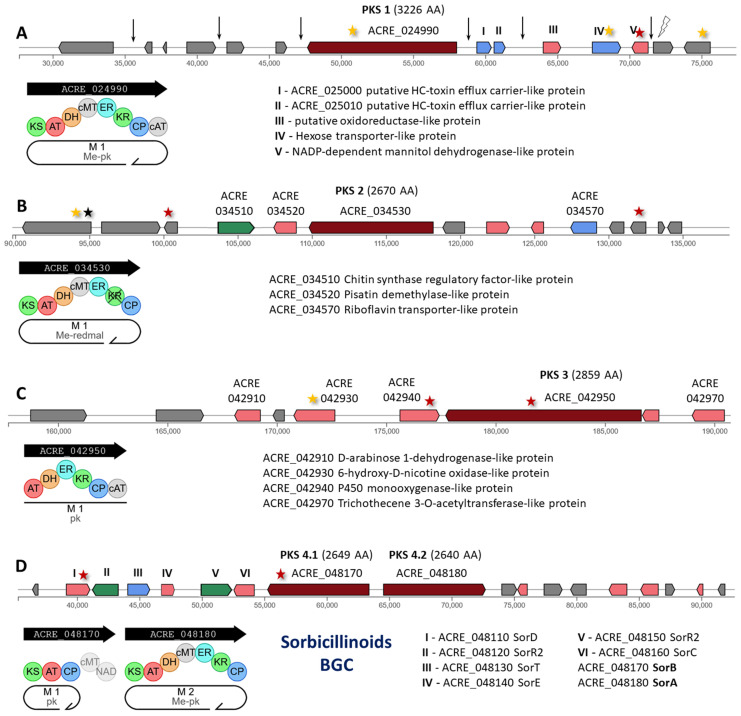
Mutational impact of the CSI program on secondary metabolite gene clusters with PKS 1–4 in *A. chrysogenum* HY: (**A**)—PKS 1, (**B**)—PKS 2, (**C**)—PKS 3, and (**D**)—PKS 4. Sequences for PKS are filled in brown boxes, biosynthetic tailoring enzymes are filled in red boxes, transporters are filled in blue boxes, path-specific regulators are filled in green boxes, and genes with unknown functions for secondary metabolite production are filled in gray boxes. Mutations in exons are indicated by asterisks filled in colors according to the following categories: HIGH—black, MODERATE—red, LOW—yellow. Arrows indicate sites for SNPs outside the exons. The arrow with lightning indicates the intron variant. Domains within PKS modules are indicated by circles with the following inscribed letters: KS—ketoacyl synthase; AT—acyltransferase; CP—acyl transfer protein (ACP); DH—dehydratase; cMT—carbon methyltransferase; ER—enoylreductase; KR—ketoreductase; cAT—choline/carnitine o-acyltransferase; and NAD—NADbinding 4 (male sterility protein).

**Figure 5:**
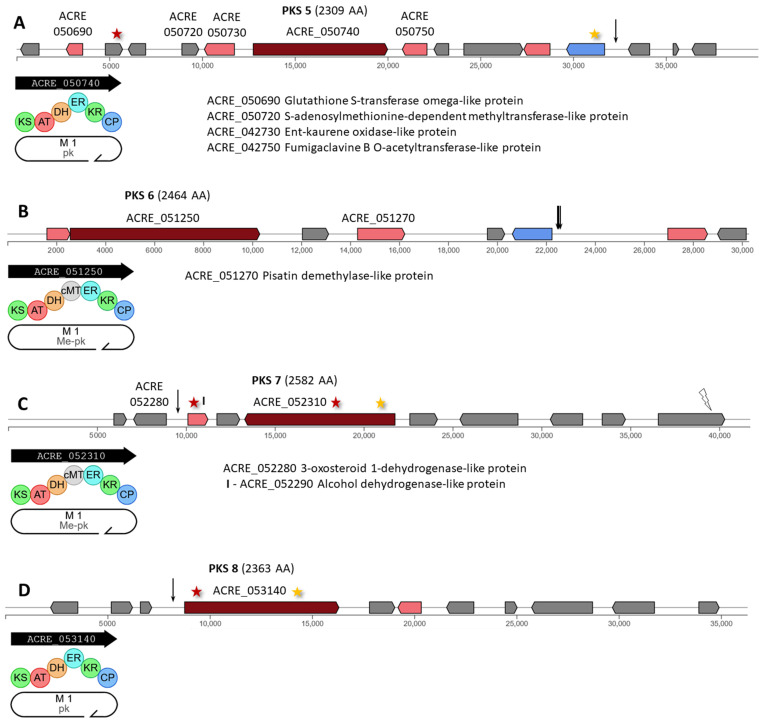
Mutational impact of the CSI program on secondary metabolite gene clusters with PKS 5–8 in *A. chrysogenum* HY: (**A**)—PKS 5, (**B**)—PKS 6, (**C**)—PKS 7, and (**D**)—PKS 8. Sequences for PKS are filled in brown boxes, biosynthetic tailoring enzymes are filled in red boxes, transporters are filled in blue boxes, and genes with unknown functions for secondary metabolite production are filled in gray boxes. Mutations in exons are indicated by asterisks filled in colors according to the following categories: MODERATE—red, LOW—yellow. Arrows indicate sites for SNPs outside the exons. The arrow with lightning indicates the intron variant. Domains within PKS modules are indicated by circles with the following inscribed letters: KS—ketoacyl synthase; AT—acyltransferase; CP—acyl transfer protein (ACP); DH—dehydratase; cMT—carbon methyltransferase; KR—ketoreductase; and ER—enoylreductase.

**Figure 6:**
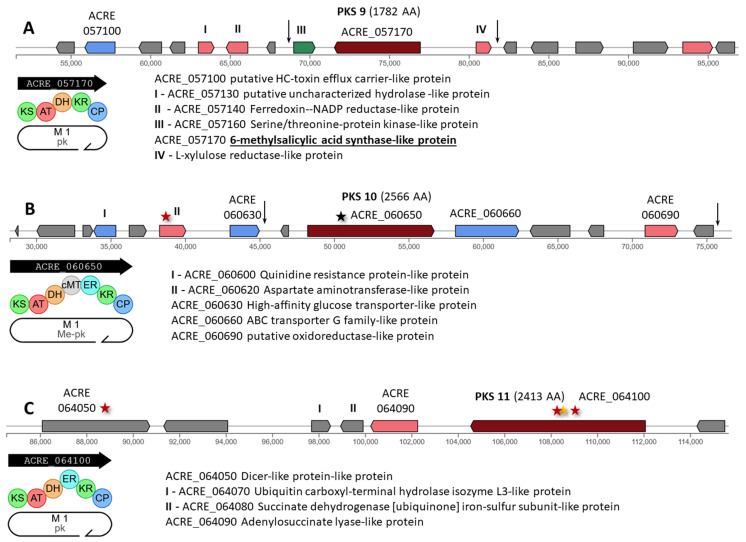
Mutational impact of the CSI program on secondary metabolite gene clusters with PKS 9–11 in *A. chrysogenum* HY: (**A**)—PKS 9, (**B**)—PKS 10, and (**C**)—PKS 11. Sequences for PKS are filled in brown boxes, biosynthetic tailoring enzymes are filled in red boxes, transporters are filled in blue boxes, path-specific regulators are filled in green boxes, and genes with unknown functions for secondary metabolite production are filled in gray boxes. Mutations in exons are indicated by asterisks filled in colors according to the following categories: HIGH—black, MODERATE—red, LOW—yellow. Arrows indicate sites for SNPs outside the exons. Domains within PKS modules are indicated by circles with the following inscribed letters: KS—ketoacyl synthase; AT—acyltransferase; CP—acyl transfer protein (ACP); DH—dehydratase; cMT—carbon methyltransferase; KR—ketoreductase; and ER—enoylreductase.

In *A. chrysogenum*, 15 of the 21 PKSs are in 14 individual clusters, with one or two (for PKS cluster 4) polyketide synthases. Mutations affected 13 out of 14 clusters with PKS of HY strain: PKS 1, 3–14 (Figure 4, Figure 5 and Figure 6). For PKS 2, four mutations were detected, located at the left (three mutations) and right (one mutation) boundaries of the cluster.

PKS 1 (ACRE_024990) encodes an iterative type I PKS of 3226 AA with carbon methyltransferase domain (Me-pk, Figure 4A). In this gene, a synonymous mutation c.6201C>T|p.Ile2067Ile occurred; in addition, one mutation each was detected in the upstream and downstream regulatory regions (Table S4). Also, synonymous mutations c.1362G>A|p.Ser454Ser in the transporter gene and c.426T>C|p.Leu142Leu in the gene with unknown function occurred; missense mutation c.469G>A|p.Val157Ile in the biosynthetic enzyme gene occurred. In addition, three more mutations occurred in the intergenic region and one in the intron region directly within this cluster; two intergenic mutations were also found in the region near the left border of the cluster.

PKS 2 (ACRE_034530) encodes an iterative type I PKS of 2660 AA (ME-redmal, Figure 4B). No mutations occurred within this cluster, but mutations in genes with unknown functions were detected near its left and right borders (Table S4). Near the left border of the cluster, mutations occurred: in the ACRE_034480 gene—frameshift variant c.95dupA|p.Ala33fs and synonymous mutation c.702G>A|p.Lys234Lys; in the ACRE_034500 gene, missense mutation c.628C>A|p.Leu210Ile. Near the right border, a missense mutation c.364G>A|p.Glu122Lys occurred in the ACRE_034590 gene.

PKS 3 (ACRE_042950) encodes type I PKS of 2859 AA (pk, Figure 4C). A missense variant, c.3409A>C|p.Lys1137Gln, was found in this transcript; this substitution may have a significant impact on PKS 3 activity (Table S4). In addition, another strong nonsynonymous substitution, c.1576C>T|p.Arg526Trp, occurred in a neighboring tailoring enzyme, cytochrome P450, encoded by the ACRE_042950. In another tailoring enzyme gene of this cluster (ACRE_042930, encodes 6-hydroxy-D-nicotine oxidase-like protein), a synonymous substitution c.606C>T|p.Leu202Leu occurred.

PKS 4 contains well-characterized sorbicillinoid BGC, including polyketide synthase genes: PKS 4.1 (ACRE_048170, encodes SorB of 2649 AA) and PKS 4.2 (ACRE_048180, encodes SorA of 2640 AA) (Figure 4D). More detailed information on the products and genes of this BGC is described in Section 2.2.1. A characteristic feature of *A. chrysogenum* HY is the complete loss during the CSI program of the characteristic yellowish-cream color [26] obtained as a result of the biosynthesis of sorbicillinoids [48]. In this context and in light of the known data on sorbicillinoid BGCs in *A. chrysogenum* WT, it was important to identify possible changes in this cluster in *A. chrysogenum* HY. It turned out that in this strain, there was a substitution of Ile for Lys in the gene for SorB, c.7190T>A|p.Ile2397Lys (Table S4). This is very important data since it has been shown that individual missense mutations in PKS can lead to complete inactivation of the production of all sorbicillinoids. Thus, in Penicillium *chrysogenum*, as a result of the functioning of the sorbicillinoid cluster, about two dozen colored compounds can be synthesized, corresponding to both the final products of this branched metabolic pathway and its intermediates [65]. However, one point mutation Leu146Phe in the SorA (Pc21g05080, PKS13) leads to transcriptional silencing of sorbicillinoid BGC [41] and loss of the characteristic yellowish pigment coloration in CSI-derived penicillin G (PenG) producer obtained from the pigmented natural isolate *P. chrysogenum* NRRL1951 [62]. Since for *A. chrysogenum* it was also shown that inactivation of *sorA* or *sorB* resulted in discoloration, a complete lack of pigmentation [48], the Ile2397Lys point mutation in the SorB (KFH44362, encoded by the gene ACRE_048170), identified in *A. chrysogenum* HY, may be the cause of inactivation of sorbicillinoid biosynthesis and loss of the characteristic yellowish color.

In addition, a point mutation c.1477C>T|p.His493Tyr was found in the tailoring enzyme BGC of sorbicillinoids SorD (KFH44377, encoded by the gene ACRE_048110), responsible for the oxidative dearomatization of dihydrosorbicillin and sorbicillin to give dihydrosorbicillinol and sorbicillinol, respectively [66]. It was shown that the biosynthesis of sorbicillinoids is regulated, including through autoinduction processes since the inactivation of *sorA* and *sorB*, and the associated inactivation of sorbicillinoids production also leads to a decrease in the expression level of the corresponding biosynthetic genes [65]. Cultivation of *P. chrysogenum* DS68530 (with SorA mutation and unable to produce sorbicillinoids) on sorbicillinoid-containing spent medium derived from the DS68530Res13 strain (SorA+) resulted in upregulation of all sorbicillinoid biosynthetic genes. In this case, mutations in SorD, leading to a decrease in the autoinducers sorbicillinoids, can lead to a decrease in the level of biosynthesis of all compounds of this class.

Thus, the two key mutations that occurred in the BGC sorbicillinoids in *A. chrysogenum* HY are related to (i) Ile2397Lys substitution in the backbone enzyme, PKS SorB, and (ii) His493Tyr substitution in the tailoring enzyme, monooxygenase SorD, important for the production of autoinducers of this metabolic pathway. This is quite interesting data, since the improved β-lactam producer Wisconsin 54-1255 and its derivatives found only two mutations in PKS, at the intradomain region of SorB and a key mutation within the KS domain of the SorA. No additional mutations were found, either within the genes of this cluster or in the intergenic regions [65].

PKS 5 (ACRE_050740) encodes an iterative type I PKS of 2309 AA (pk, Figure 5A). In this cluster, a missense mutation c.633G>T|p.Lys211Asn occurred in ACRE_050700, encoding a protein of unknown function (Table S4). A synonymous substitution c.408A>G|p.Val136Val occurred in the ACRE_050790 gene for transport protein and a point mutation in the intergenic region ACRE_050790-ACRE_050800|||n.32965C>T.

PKS 6 (ACRE_051250) encodes an iterative type I PKS of 2464 AA with carbon methyltransferase domain (Me-pk, Figure 5B). This cluster revealed a series of differences in the upstream region of the transport gene ACRE_051290 (Table S4).

PKS 7 (ACRE_052310) encodes an iterative type I PKS of 2582 AA with carbon methyltransferase domain (Me-pk, Figure 5C). This gene has been mutagenized and carries a missense mutation c.3005A>C|p.Tyr1002Ser and a synonymous substitution c.1068G>A|p.Ala356Ala (Table S4). Mutations also occurred in the tailoring enzyme encoded by ACRE_052290 in the coding region (missense variant c.284C>T|p.Thr95Ile) and in the upstream region (c.-204T>A).

PKS 8 (ACRE_053140) encodes an iterative type I PKS of 2365 AA (pk, Figure 5D). In this gene, as in the gene for PKS 7, one non-synonymous (c.287T>C|p.Leu96Pro) and one synonymous (c.5346T>A|p.Ile1782Ile) substitution occurred (Table S4). In addition, a single mutation c.-145C>T occurred in the upstream region of this gene.

PKS 9 (ACRE_057170) encodes an iterative type I PKS of 1782 AA (pk, Figure 6A). This cluster contains a mutation in the upstream region of ACRE_057160, a regulatory protein gene (Table S4). Mutation n.81686T>G was also found in the intergenic region ACRE_057180-ACRE_057190.

PKS 10 (ACRE_060650) encodes an iterative type I PKS of 2566 AA with carbon methyltransferase domain (Me-pk, Figure 6B). At the beginning of this gene, a stop gained mutation c.2097T>A|p.Tyr699* occurred, leading to the appearance of a shortened product with 699 of 2566 AA and a loss of function of both the megasynthase itself and the entire PKS 10 cluster (Table S4). In addition, a missense mutation c.263T>C|p.Ile88Thr occurred in the ACRE_060620 tailoring enzyme gene; a mutation in the downstream region of the ACRE_060630 transporter gene; and another mutation in the intergenic region near the right border of the cluster.

PKS 11 (ACRE_064100) encodes an iterative type I PKS of 2413 AA (pk, Figure 6C). This gene has undergone significant mutagenic effects since three mutations have arisen in its coding region: missense c.3139G>A|p.Asp1047Asn, missense c.3605G>T|p.Gly1202Val, and synonymous c.3606A>T|p.Gly1202Gly (Table S4). A missense mutation c.2447A>T|p.Tyr816Phe in the ACRE_064050 gene also arose in this cluster.

**Figure 7:**
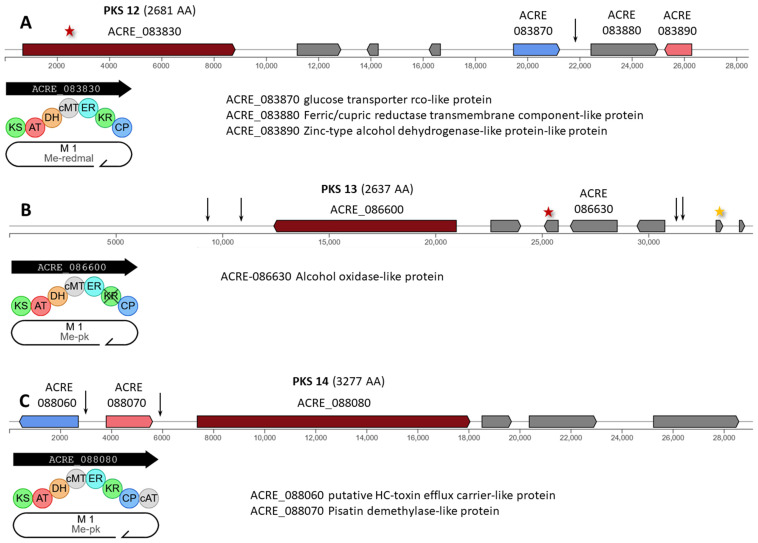
Mutational impact of the CSI program on secondary metabolite gene clusters with PKS 12–14 in *A. chrysogenum* HY: (**A**)—PKS 12, (**B**)—PKS 13, and (**C**)—PKS 14. Sequences for PKS are filled in brown boxes, biosynthetic tailoring enzymes are filled in red boxes, transporters are filled in blue boxes, and genes with unknown functions for secondary metabolite production are filled in gray boxes. Mutations in exons are indicated by asterisks filled in colors according to the following categories: MODERATE—red, LOW—yellow. Arrows indicate sites for SNPs outside the exons. Domains within PKS modules are indicated by circles with the following inscribed letters: KS—ketoacyl synthase; AT—acyltransferase; CP—acyl transfer protein (ACP); DH—dehydratase; cMT—carbon methyltransferase; KR—ketoreductase; ER—enoylreductase; and cAT—choline/carnitine o-acyltransferase.

PKS 12 (ACRE_083830) encodes an iterative type I PKS of 2681 AA (ME-redmal, Figure 7A). A missense mutation c.1372C>T|p.Arg458Cys occurred at the beginning of this gene (Table S4). Also, for this cluster, mutation n.17811G>A is observed in the intergenic region ACRE_083860-ACRE_083870.

PKS 13 (ACRE_086600) encodes an iterative type I PKS of 2637 AA with carbon methyltransferase domain (Me-pk, Figure 7B). In BGC with this enzyme, mutations occurred in the genes ACRE_086620 (missense mutation c.533T>A|p.Ile178Lys) and ACRE_086650 (synonymous variant c.60T>C|p.Ile20Ile) (Table S4). In addition, two mutations were found in the intergenic regions of the left and right parts of the cluster.

PKS 14 (ACRE_088080) encodes an iterative type I PKS of 3277 AA with carbon methyltransferase domain (Me-pk, Figure 7C). In this cluster, two mutations belonging to the MODIFIER category occurred: c.-163T>A upstream gene ACRE_088060 for transporter and c.*89C>A downstream gene ACRE_088070 for tailoring enzyme (Table S4).

#### 2.3.3. Mutations in BGCs with TPC in *A. chrysogenum* HY

**Figure 8:**
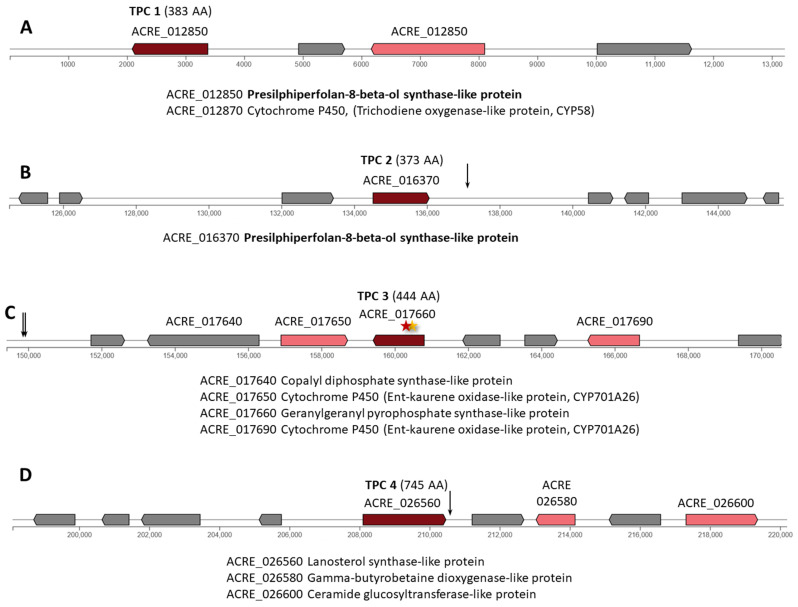
Mutational impact of the CSI program on secondary metabolite gene clusters with TPC (terpene cyclase) 1–4 in *A. chrysogenum* HY: (**A**)—TPC 1, (**B**)—TPC 2, (**C**)—TPC 3, and (**D**)—TPC 4. Sequences for TPC are filled in brown boxes, biosynthetic tailoring enzymes are filled in red boxes, and genes with unknown functions for secondary metabolite production are filled in gray boxes. Mutations in exons are indicated by asterisks filled in colors according to the following categories: MODERATE—red, LOW—yellow. Arrows indicate sites for SNPs outside the exons.

**Figure 9:**
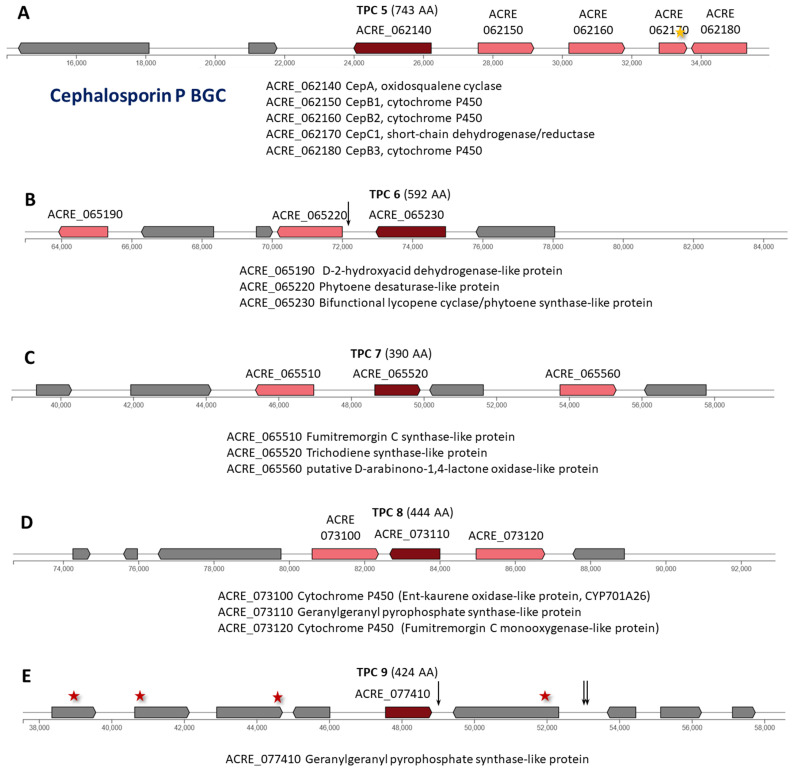
Mutational impact of the CSI program on secondary metabolite gene clusters with TPC (terpene cyclase) 5–9 in *A. chrysogenum* HY: (**A**)—TPC 5, (**B**)—TPC 6, (**C**)—TPC 7, (**D**)—TPC 8, and (**E**)—TPC 9. Sequences for TPC are filled in brown boxes, biosynthetic tailoring enzymes are filled in red boxes, and genes with unknown functions for secondary metabolite production are filled in gray boxes. Mutations in exons are indicated by asterisks filled in colors according to the following categories: MODERATE—red, LOW—yellow. Arrows indicate sites for SNPs outside the exons.

In *A. chrysogenum*, 9 of 11 TPC are in individual clusters. In the HY strain, mutations affected 6 of the 9 TPC clusters (TPC 2–6 and 9), which is lower than for the PKS clusters (where mutations affected 93% of clusters) and is comparable with NRPS clusters (78% of clusters affected by mutations). No mutations were detected in clusters: TPC 1 (ACRE_012850) encodes a 383 AA enzyme (Figure 8A); TPC 7 (ACRE_065520) encodes a 390 AA enzyme (Figure 9C); and TPC 8 (ACRE_073110) encodes a 444 AA enzyme (Figure 9D).

TPC 2 (ACRE_016370) encodes a 373 AA enzyme annotated as pre-silphiperfolan-8-beta-ol synthase-like protein (Figure 8B). In the downstream regulatory region of this gene (intergenic region ACRE_016380-ACRE_016390 n.137121A>C), a single mutation occurred for the entire cluster (Table S4).

TPC 3 (ACRE_017660) encodes a 444 AA enzyme annotated as geranylgeranyl pyrophosphate synthase-like protein (Figure 8C). Two mutations occurred in this gene: missense (c.536C>T|p.Ser179Phe) and synonymous (c.529C>T|p.Leu177Leu) (Table S4). Your mutations are also identified in the intergenic region in the area near the left border of the cluster.

TPC 4 (ACRE_026560) encodes a 745 AA enzyme (Figure 8D). In the downstream regulatory region of this gene, the mutation c.*251T>A was found, the only one in this cluster.

TPC 5 contains a BGC for the synthesis of cephalosporins P and the major component of the fraction of these compounds, cephalosporin P_1_, in particular (Figure 9A) [46]. The components of this cluster are described in more detail in Section 2.2.1. No mutations occurred in the ACRE 026560 gene for the backbone enzyme oxidosqualene cyclase CepA of 745 AA. The only mutation in this cluster is categorized as LOW and occurs in the ACRE_062170 gene (synonymous variant c.474C>T|p.Ala158Ala), encoding CepC1, a short-chain dehydrogenase/reductase (Table S4).

TPC 6 (ACRE_065230) encodes a 592 AA enzyme (Figure 9B). The only mutation in this cluster occurred in the intergenic region ACRE_065220-ACRE_065230, n.72152G>A (Table S4).

The greatest number of mutational changes among BGCs with individual TPCs occurred in TPC 9, containing the ACRE_077410 gene encoding a 424 AA backbone enzyme (Figure 9E). Four nonsynonymous substitutions occurred in four genes of this cluster: c.468A>T|p.Lys156Asn in gene ACRE_077370; c.8C>T|p.Ser3Phe in gene ACRE_077380; c.1375G>A|p.Glu459Lys in gene ACRE_077390; and c.350A>G|p.Lys117Arg in gene ACRE_077420 (Table S4). In addition, a mutation appeared in the intergenic region with the central gene for TPC (n.49094A>G in the intergenic region ACRE_077410-ACRE_077420), and two mutations in the intergenic region ACRE_077420-ACRE_077430.

#### 2.3.4. Mutations in Hybrid BGCs in *A. chrysogenum* HY

**Figure 10:**
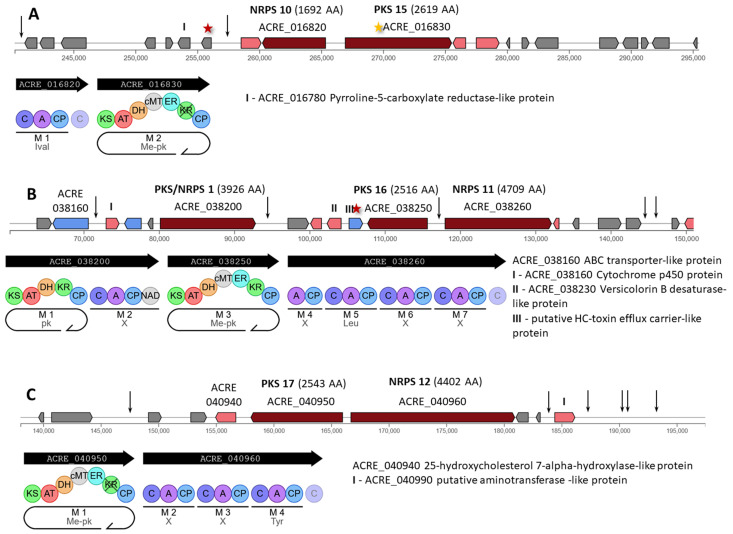
Mutational impact of the CSI program on hybrid biosynthetic gene clusters (BGCs) 1–3 in *A. chrysogenum* HY: (**A**)—hybrid BGC 1, (**B**)—hybrid BGC 2, and (**C**)—hybrid BGC 3. Sequences for backbone enzymes are filled in brown boxes, biosynthetic tailoring enzymes are filled in red boxes, transporters are filled in blue boxes, and genes with unknown functions for secondary metabolite production are filled in gray boxes. NRPS—non-ribosomal peptide synthetase; PKS—polyketide synthase. Mutations in exons are indicated by asterisks filled in colors according to the following categories: MODERATE—red, LOW—yellow. Arrows indicate sites for SNPs outside the exons. Domains within NRPS and PKS modules are indicated by circles with the following inscribed letters: A—adenylation; CP_NRPS_—peptidyl carrier protein (PCP); C—condensation; KS—ketoacyl synthase; AT—acyltransferase; CP_PKS_—acyl transfer protein (ACP); DH—dehydratase; cMT—carbon methyltransferase; KR—ketoreductase; ER—enoylreductase; and NAD—NADbinding 4 (male sterility protein).

**Figure 11:**
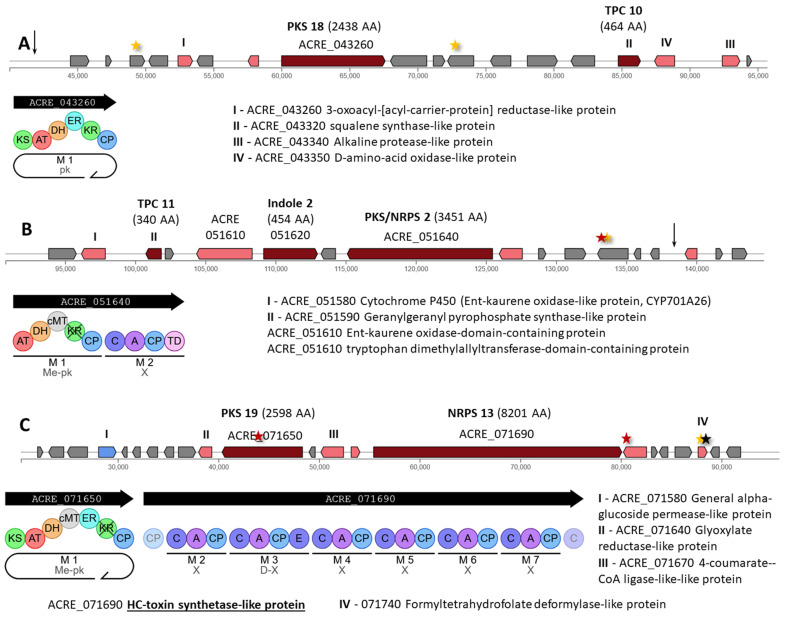
Mutational impact of the CSI program on hybrid biosynthetic gene clusters (BGCs) 4–6 in *A. chrysogenum* HY: (**A**)—hybrid BGC 4, (**B**)—hybrid BGC 5, and (**C**)—hybrid BGC 6. Sequences for backbone enzymes are filled in brown boxes, biosynthetic tailoring enzymes are filled in red boxes, transporters are filled in blue boxes, and genes with unknown functions for secondary metabolite production are filled in gray boxes. NRPS—non-ribosomal peptide synthetase; PKS—polyketide synthase; TPC—terpene cyclase. Mutations in exons are indicated by asterisks filled in colors according to the following categories: HIGH—black, MODERATE—red, LOW—yellow. Arrows indicate sites for SNPs outside the exons. Domains within NRPS and PKS modules are indicated by circles with inscribed letters: A—adenylation; CP_NRPS_—peptidyl carrier protein (PCP); C—condensation; TD—terminal reductase; KS—ketoacyl synthase; AT—acyltransferase; CP_PKS_—acyl transfer protein (ACP); DH—dehydratase; cMT—carbon methyltransferase; ER—enoylreductase; and KR—ketoreductase.

**Figure 12:**
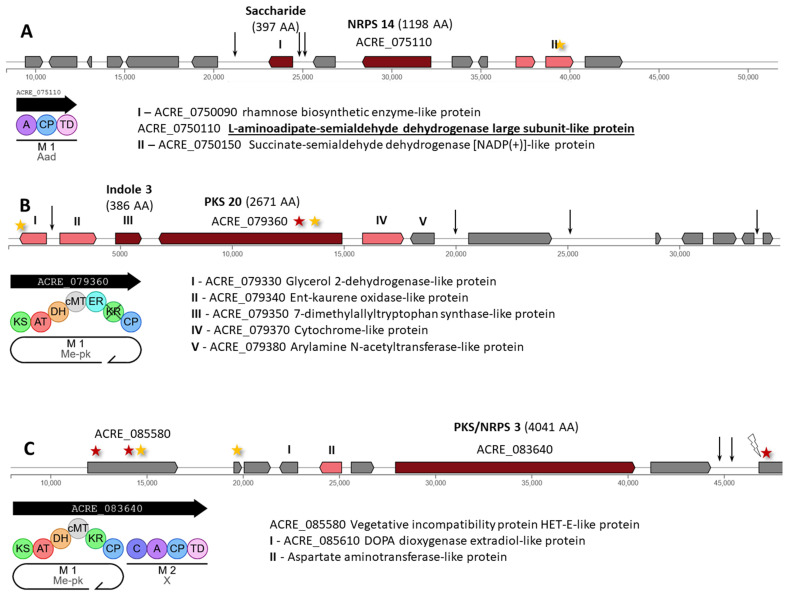
Mutational impact of the CSI program on hybrid biosynthetic gene clusters (BGCs) 7–9 in *A. chrysogenum* HY: (**A**)—hybrid BGC 7, (**B**)—hybrid BGC 8, and (**C**)—hybrid BGC 9. Sequences for backbone enzymes are filled in brown boxes, biosynthetic tailoring enzymes are filled in red boxes, and genes with unknown functions for secondary metabolite production are filled in gray boxes. NRPS—non-ribosomal peptide synthetase; PKS—polyketide synthase. Mutations in exons are indicated by asterisks filled in colors according to the following categories: MODERATE—red, LOW—yellow. Arrows indicate sites for SNPs outside the exons. The arrow with lightning indicates the intron variant. Domains within NRPS and PKS modules are indicated by circles with the following inscribed letters: A—adenylation; CP_NRPS_—peptidyl carrier protein (PCP); C—condensation; TD—terminal reductase; KS—ketoacyl synthase; AT—acyltransferase; CP_PKS_—acyl transfer protein (ACP); DH—dehydratase; cMT—carbon methyltransferase; ER—enoylreductase; and KR—ketoreductase.

In *A. chrysogenum* HY, at least one mutation occurred in each of the nine hybrid clusters (Figure 10, Figure 11 and Figure 12).

The Hybrid 1 cluster contains NRPS 10 (ACRE_016820), encoding a single-modular enzyme of 1692 AA, and PKS 15 (ACRE_016830), encoding an iterative type I PKS of 2319 AA with carbon methyltransferase domain (Me-pk, Figure 10A). A synonymous mutation c.2694G>A|p.Lys898Lys occurred in the PKS 15 gene (Table S4). Also, a nonsynonymous substitution, c.77G>A|p.Arg26Gln, occurred in the ACRE_016800 gene. In addition, mutations appeared in the intergenic region ACRE_016800-ACRE_016810 (n.257626C>) and in the intergenic region near the left border of the cluster.

The Hybrid 2 contains three megasynthases: (i) PKS/NRPS 1 (ACRE_038200), encoding a 3926 AA fusion between an iterative type I PKS and a single-module NRPS; (ii) PKS 16 (ACRE_038250), encoding an iterative type I PKS of 2516 AA with carbon methyltransferase domain (Me-pk); (iii) and NRPS 11 (ACRE_038260), encoding a four-module enzyme of 4709 AA (Figure 10B). In the central region of this cluster, a missense mutation c.1532C>T|p.Ser511Phe occurred in the ACRE_038240 gene for putative HC-toxin efflux carrier-like protein (Table S4). There were also five mutations in the regulatory regions of genes: in the intergenic region ACRE_038160-ACRE_038170 (n.71858C>T); in the intergenic region ACRE_038200-ACRE_038210 (n.95486T>A); in the bidirectional promoter region for PKS 16 and NRPS 11 (n.117768T>C); and two mutations (n.145651G>A and n.147533delA) in the intergenic region ACRE_038300-ACRE_038310.

The Hybrid 3 cluster contains PKS 17 (ACRE_040950), encoding an iterative type I PKS of 2543 AA with carbon methyltransferase domain (Me-pk), and NRPS 12 (ACRE_040960), encoding a three-module enzyme of 4402 AA (Figure 10C). In this cluster, six mutations occurred outside the coding regions of genes: in the intergenic region ACRE_040910-ACRE_040920 (n.147207delT); upstream gene ACRE_040980 encoding a tailoring enzyme; and four mutations in the intergenic region ACRE_040990-END (n.187617C>A, n.190502A>G, n.190904A>G, n.193032A>G) (Table S4).

The Hybrid 4 cluster contains PKS 18 (ACRE_043260), encoding an iterative type I PKS of 2438 AA, and TPC 10 (ACRE_043320), encoding a 454 AA enzyme (Figure 11A). Overall, this cluster was slightly affected by mutagenesis, as two synonymous substitutions arose in the genes ACRE_043210 (c.423G>A|p.Gln141Gln) and ACRE_043290 (c.1485C>A|p.Gly495Gly), as well as one mutation in the intergenic region ACRE_043180-ACRE_043190 (n.43299C>T) (Table S4).

Three backbone enzymes are assembled in the Hybrid 5 cluster: (i) TPC 11 (ACRE_051590), encoding a 454 AA enzyme; (ii) Indole 2 (ACRE_051620), encoding an 804 AA fusion protein with cytochrome P450 and tryptophan dimethylallyltransferase domains for the biosynthesis of indoles; and (iii) PKS/NRPS 2 (ACRE_051640), encoding a 3451 AA fusion between an iterative type I PKS with carbon methyltransferase domain (Me-pk) and a single-module NRPS (Figure 11B). In this cluster, two mutations occurred in the gene of unknown function ACRE_051680 (missense c.1390C>T|p.Pro464Ser and synonymous c.1398C>T|p.Val466Val) and in the downstream region of the gene ACRE_051710 (c.*792G>A) encoding a tailoring enzyme.

The Hybrid 6 cluster contains PKS 19 (ACRE_071650), encoding an iterative type I PKS of 2598 AA with carbon methyltransferase domain (Me-pk), and NRPS 13 (ACRE_071690), encoding a six-module enzyme of 8201 AA (Figure 11C). This cluster was heavily mutagenized in the CSI program, with mutations in the MODERATE and HIGH categories occurring in genes encoding backbone and tailoring enzymes. Thus, in the ACRE_071650 gene for PKS 19, a missense mutation c.4420C>A|p.Leu1474Ile arose (Table S4). In addition, in the tailor’s gene ACRE_071700, a missense mutation c.1556G>A|p.Gly519Glu appeared; this substitution simultaneously affects the downstream region for the NRPS 13 gene (and leads to a change c.*529C>T). In the ACRE_071740 gene for the tailoring enzyme, a stop lost and splice region variant arose, resulting in c.896A>C|p.Ter299Serext*?|896/897|896/897|299/298. In addition, a synonymous substitution c.894C>T|p.Ala298Ala occurred in this gene.

The Hybrid 7 cluster contains two backbone enzymes: ACRE_0750090 for the 397 AA rhamnose biosynthetic enzyme-like protein and NRPS 14 (ACRE_075110), encoding a single-modular enzyme of 1198 AA (Figure 12A). In this cluster, a synonymous mutation c.693G>A|p.Lys231Lys arose in the ACRE_075150 gene encoding a tailoring enzyme (Table S4). Three mutations outside the coding regions were also identified: one mutation in the intergenic region ACRE_075080-ACRE_075090 (n.22531T>C) and two mutations in the intergenic region ACRE_075090-ACRE_075100 (n.24964C>T and n.25507T>C), affecting the upstream regulatory region of the central gene for saccharide synthesis.

The Hybrid 8 cluster contains Indole 3 (ACRE_051620), encoding a 7-dimethylallyltryptophan synthase-like protein of 386 AA, and PKS 20 (ACRE_079360), encoding an iterative type I PKS of 2671 AA with carbon methyltransferase domain (Me-pk) (Figure 12B). Mutations affected the central gene PKS 20 as follows: missense c.1780T>C|p.Phe594Leu and synonymous c.1329C>T|p.Gly443Gly (Table S4). Another synonymous substitution, c.849T>C|p.Ile283Ile, occurred at the end of the ACRE_079330 gene encoding a tailoring enzyme. In the upstream region of this gene, a mutation c.-361T>A also occurred. In the intergenic region located near the right border of the cluster, three more mutations arose: n.20296A>G in the ACRE_079380-ACRE_079390 region, n.25913C>A in the ACRE_079390-ACRE_079400 region, and n.33388delG in the ACRE_079430-ACRE_079440 region.

The Hybrid 9 cluster contains PKS/NRPS 3 (ACRE_083640), encoding a 4041 AA fusion between an iterative type I PKS with carbon methyltransferase domain (Me-pk) and a single-module NRPS (Figure 11B). The most significant changes occurred in the ACRE_085580 gene with unknown function: missense variants c.1682C>T|p.Pro561Leu and c.118G>A|p.Asp40Asn; and synonymous variant c.1785C>T|p.Phe595Phe (Table S4). Also, a synonymous substitution occurred in the ACRE_085590 gene (c.1563C>T|p.Ile521Ile). The following mutations occurred in the ACRE_085660 gene: in the coding region—missense mutation c.1025T>A|p.Ile342Asn, in the intron—mutation c.543+19G>A, in the upstream regulatory region—mutations c.-975T>C and c.-973A>G, in its intergenic region—mutation n.1917C>T.

### 2.4. Key Mutations in A. chrysogenum HY

*A. chrysogenum* HY carries 56 mutations that fall into the HIGH category and lead in most cases to a complete loss of function of the product (Tables S1 and S5). From this point of view, some mutations from the MODERATE category, which includes non-synonymous substitutions, can also lead to a complete loss of function of the encoded protein, as happened with SorA in improved producers of *P. chrysogenum*, where the Leu146Phe point mutation led to a complete inactivation of sorbicillinoids biosynthesis [62]. However, assessing the contribution of non-synonymous substitutions in silico is significantly more difficult and, in most cases, requires experimental evidence. In this regard, the current work was limited to consideration of mutations from the HIGH category.

A total of 5 of the 56 HIGH mutations affected BGC as follows: megasynthases NRPS 4 (ACRE_012290) and PKS 10 (ACRE_060650), two biosynthetic enzymes (ACRE_071740, annotated as formyltetrahydrofolate deformylase-like protein in the Hybrid 6 cluster and ACRE_034480, annotated as UDP-glucose:glycoprotein glucosyltransferase-like protein) and one transcript of unknown function ACRE_003300 (in the right border region of NRPS 1 for the “early” beta-lactam cluster and in PKS 2) (Table S4). In total, the function is unknown for 17 and 56 transcripts with HIGH mutations (Table S5).

#### 2.4.1. Mutations in Biosynthetic Genes

Mutations in the HIGH category affected various metabolic processes (Table 3). It is possible that functions that allow the organism to adapt to various environmental conditions disappeared, but additional sources were released to intensify the target metabolism of cephalosporin C. Thus, the ACRE_051330 gene disruption occurred for succinyl-CoA--L-malate CoA-transferase beta subunit-like protein (stop gained, c.1355C>A|p.Ser452*), involved in the 3-hydroxypropionate cycle used for autotrophic carbon dioxide fixation. There was also inactivation of the ACRE_072830 gene for aldehyde dehydrogenase-like protein (stop gained, c.452C>A|p.Ser151*), which belongs to the superfamily of enzymes that are critical for certain life processes. Also, the biosynthetic repertoire of *A. chrysogenum* HY appears to have been reduced by frameshifts in two genes: ACRE_025660 (mutation c.454delC| p.Leu152fs) for putative epoxide hydrolase-like protein and ACRE_061140 (mutation c.980delA| p.Tyr327fs) for cytochrome P450, annotated as pisatin demethylase-like protein. Stop lost mutation c.896A>C| p.Ter299Serext*? in the ACRE_071740 gene apparently disrupts the function of the encoded formyltetrahydrofolate deformylase-like protein. This enzyme is capable of hydrolyzing carbon-nitrogen bonds other than peptide bonds, such as in linear amides, and is involved in various metabolic pathways such as one carbon pool by folate, dicarboxylate, and glyoxylate metabolism. The listed mutations in metabolic enzymes could lead to the slower growth rate observed for *A. chrysogenum* HY [26].

Several mutations affected lipid metabolism. A nonsense mutation in the ACRE_067920 gene (c.979C>T|p.Arg327*) results in a loss of function of the encoded esterase-like protein. This protein contains the “Aes” (acetyl/esterase/lipase) region responsible for lipid transport and metabolism, as well as the “abhydrolase” (alpha/beta hydrolases) region. Disruption in the ACRE_009560 gene (frameshift c.44dupA|p.Asp15fs) results in inactivation of peroxisomal 2,4-dienoyl-CoA reductase, which plays a key role in (poly)unsaturated fatty acid oxidation via metabolizing 2,4-dienoyl-CoA in the peroxisome to 3-enoyl-CoA in a NADPH-dependent process [67,68]. As a result, *A. chrysogenum* HY may have a disturbance in the utilization of fatty acids, but on the other hand, there may be an “unloading” of the biosynthetic reactions of primary metabolism in peroxisomes in order to intensify the reactions of the cephalosporin C biosynthesis pathway, where a series of re-reactions occur for the isomerization of isopenicillin N to penicillin N [69]. In addition to mutations that can cause disturbances in lipid metabolism as such, mutations affecting phospholipid metabolism have been detected. Thus, a nonsense mutation c.26T>G|p.Leu9* was detected in the ACRE_090470 gene encoding putative CDP-alcohol phosphatidyl-transferase class-I family protein-like protein, which catalyzes an essential reaction for phospholipid biosynthesis, which are critical components of cell membranes. A nonsense mutation c.1093G>T|p.Glu365* was also detected in the ACRE_084300 gene, affecting glycerolipid metabolism. This occurs because the encoded phospholipid:diacylglycerol acyl-transferase-like protein, disrupted by mutation, catalyzes the acyl-CoA-independent formation of triacylglycerol. This protein is capable of converting membrane lipids into triacylglycerol during nitrogen starvation stress in some organisms [70]. It is evident that in *A. chrysogenum* HY, the variability of lipid metabolism reactions has generally decreased. The role of these changes is difficult to assess due to the complexity of lipid metabolism and the unspecified roles of the inactivated enzymes. Another nonsense mutation occurred in the ACRE_084300 gene (c.1093G>T|p.Glu365*), containing the Galactosyltransferase region, which catalyzes the transfer of galactose, which can be used in numerous cellular reactions.

A series of mutations that occurred in *A. chrysogenum* HY led to the inactivation of nucleotide metabolism enzymes. In particular, a nonsense mutation in the ACRE_070590 gene (c.280C>T|p.Gln94*) leads to the inactivation of NAD(P)H-hydrate epimerase-like protein. This enzyme converts (R)-NAD(P)HX (a damaged form of NAD(P)H resulting from enzymatic or heat-dependent hydration) to the corresponding (S) forms, which could then be repaired to active dinucleotides by ATP-dependent NAD(P)H-hydrate dehydratase. A frameshift mutation in the first codon of the ACRE_007400 gene (c.1dupA|p.Met1fs) leads to loss of function of the hypothetical protein with NUDIX Hydrolase region, responsible for the hydrolysis of NUcleoside DIphosphates linked to other moieties, X. Members of this family are recognized by the highly conserved 23-residue Nudix motif (GX5EX7REUXEEXGU, where U = I, L, or V), which forms a structural motif that functions as a metal-binding and catalytic site. Substrates of Nudix hydrolase include intact and oxidatively damaged dinucleotide cofactors, nucleoside triphosphates, dinucleoside polyphosphates, etc. These mutations potentially reduce the ability of *A. chrysogenum* HY to repair damage and may have had some role in the increase in mutation rates in the CSI program. On the other hand, the fungal Nudix hydrolase may be a phytopathogenicity factor that is not required for the production of the target metabolite in the improved strain [71].

Several mutations that occurred could reduce S-adenosyl-L-methionine (SAMe) uptake in *A. chrysogenum* HY. In particular, one of the key reactions that remove SAMe from the pool for methylation is associated with its conversion to 1-aminocyclopropane-1-carboxylicacid (ACC) by the enzyme 1-aminocyclopropane-1-carboxylate synthase (ACC-synthase, ACS) [72,73]. From the intermediate compound ACC, ethylene, jasmonyl-ACC, γ-glutamyl-ACC, and α-ketobutyrate can then be synthesized [72]. *A. chrysogenum* HY has a frameshift mutation c.1107_1110dupCCCG|p.Lys371fs in the ACRE_065750 gene encoding 1-aminocyclopropane-1-carboxylate synthase-like protein-like protein. It can be assumed that this strain has lost the ability to consume SAMe for the synthesis of ACC and its derivatives, such as ethylene. Another mutation occurred in the ACRE_081590 gene (nonsense mutation c.19633C>T|p.Gln6545*), encoding 4-dimethylallyltryptophan N-methyltransferase-like protein. This enzyme is required for the biosynthesis of indole alkaloids and uses SAMe as a substrate. Inactivation of this enzyme likely not only disrupts alkaloid biosynthesis, which competes for resources with the target biosynthesis of cephalosporin C but also reduces the load on the SAMe pool.

Biosynthetic gene clusters were significantly mutagenized (which is described in detail in Section 2.3), but mutations classified as HIGH occurred in only two (out of 53 found in silico) backbone enzymes. Among backbone enzymes, the frequency of HIGH mutations is 3.7%, while the genome average is 0.63% (56 HIGH mutations among 8901 annotated protein-coding sequences, [53]). In *A. chrysogenum* HY, NRPS 4 (as a result of nonsense mutation c.19633C>T|p.Gln6545* in the ACRE_012290 gene) and PKS 10 (as a result of nonsense mutation c.2097T>A|p.Tyr699* in the ACRE_060650 gene) are inactivated.

Since the CSI program involved mutational selection, most mutations that arose in biosynthetic genes are associated with secondary cell needs. Their disruption does not significantly affect the cell’s vital functions and can potentially free up resources for the needs of the target metabolism.

#### 2.4.2. Mutations in Transporter Genes

Mutations belonging to the HIGH category occurred in five genes of *A. chrysogenum* HY transporters, one of them (ACRE_087850) encodes P-type Na^+^-ATPase, and the remaining four encode multidrug (MDR) transporters (Table 4).

Transcript ACRE_087850 encodes 1142 AA product, annotated as calcium-transporting ATPase-like protein. This sequence has ~83% identity with the P-type Na^+^-ATPase from *Metarhizium* sp. or *Fusarium* sp. The nonsense mutation 1577G>A|p.Trp526* that occurred in *A. chrysogenum* HY leads to the formation of a product that is shortened by more than two times and to a complete loss of function since the shortened product lacks an active center. Loss of this transporter potentially reduces the consumption of intracellular ATP, which can be used to produce the target beta-lactam metabolite, which is accompanied by ATP consumption [31].

There were also HIGH mutations in the genes of MDR transporters, belonging to three (out of six) distinguished classes: (i) ABC transporter (ACRE_075010, splice donor and intron variant c.3159+1G>A); (ii) MFS transporters (ACRE_057310, frameshift c.416_420delTGGGA|p.Val139fs and ACRE_072140, stop lost and splice region 1300T>C| p.Ter434Glnext*?); and (iii) MATE transporter (ACRE_045230, splice acceptor and intron variant c.1054-2A>G). Such disruptions in MDR transporter genes could also facilitate the release of cellular energy resources, such as ATP (in the case of ABC transporter disruption) and electrochemical gradient of protons (in the case of MFS and MATE transporter disruptions) to support the intensified target metabolism of cephalosporin C.

#### 2.4.3. Mutations in Regulatory Genes

Mutations from the HIGH category also led to the inactivation of a number of regulatory proteins (Table 5). The first group of regulators studied included putative transcription factors. In the ACRE_051010, a frameshift mutation (c.483dupA|p.Val162fs) resulted in the inactivation of a putative regulator containing bZIP: DNA binding and dimerization domain. A nonsense mutation c.373A>T|p.Lys125 also occurred in the ACRE_021680 gene, encoding a hypothetical protein that shares more than 72% identity with transcriptional activators from *Fusarium longipes*, *F. langsethiae*, and *F. flagelliforme*. The direct role of these disturbances in *A. chrysogenum* HY is difficult to assess, other than that they cause changes at the transcriptional level. On the other hand, in the ACRE_072410, a nonsense mutation c.1910C>A|p.Ser637* occurred, apparently leading to a loss of function of this 1116 AA protein containing the Zinc finger C-x8-C-x5-C-x3 -H type motif. C3H1 ZnFs are thought to bind to unstructured RNAs [74,75], often with a preference for AU-rich sequences, and control mRNA decay [76,77]. In addition, this putative protein contains a region encoding the transcriptional regulator ICP4. The nonsense mutation c.55G>T|p.Glu19* in the ACRE_088010 gene resulted in a truncation of up to 19 (out of 793) AA of the putative R3H domain-containing protein, which in addition to this domain also contains the ICP4 region, SUZ domain, and C-terminal FtsK domain. The overall role of this inactivated multifunctional protein is unclear, but its individual regions have been characterized. The R3H (RxxxH) motif is so named because it contains invariant arginine and a conserved histidine, which are separated by three residues; it binds ssDNA or ssRNA in a sequence-specific manner. ICP4 region иcпoльзyeтcя для тpaнcкpипциoннoй aктивaции; SUZ domain иcпoльзyeтcя for RNA-binding, в тoм чиcлe on nonsense-mediated decay target transcripts [78], FtsK is double-stranded DNA translocase, interacts with other cell division proteins, and regulates Xer-mediated recombination [79]. Perhaps a disruption in machinery such as ACRE_088010 allows target transcripts to escape “quality control” at the transcriptional level, which speeds up their uptake in a highly active producer.

A nonsense mutation in the ACRE_043640 gene (c.1148T>A|p.Leu383*) and a frameshift mutation in the ACRE_078410 gene (c.450delC|p.Ile151fs) resulted in the inactivation of two corresponding serine/threonine protein kinase-like proteins. Such changes could have a wide range of effects, since protein kinases, as a result of phosphorylation of amino acid residues, are capable of changing enzymatic activity, affecting metabolic pathways and signal transmission, regulating the cell cycle and differentiation, etc. In addition, a nonsense mutation c.2401C>T|p.Gln801* occurred in the ACRE_032470 gene of the Scy1-type pseudokinase domain-containing protein of 986 AA. Typically, pseudokinases lack one or more canonical amino acid residues required for interaction with ATP and metal ions, which is necessary for the phosphorylation of substrates. However, these proteins are important for regulation as dynamic scaffolds, competitors, or modulators of protein–protein interactions [80]. The ACRE_045800 gene encodes a 1216 AA stress response-like protein. This protein contains the NST1 region for suppressing salt tolerance; the Bin/Amphiphysin/Rvs (BAR) domain, a dimerization module for membrane binding and membrane curvature detection; the XRCC4 region for repairing double-strand DNA breaks and the V(D)J recombination protein XRCC4; and other regions. The frameshift mutation c.2228delA|p.Lys743fs appears to result in a significant loss of function of this multifunctional regulatory protein or completely inactivates it.

A series of mutations affected factors used by the fungal cell to regulate the cell cycle and division. The nonsense mutation c.777G>A|p.Trp259* occurred in the ACRE_084130 gene for meiotically up-regulated gene 80 protein-like. This protein is involved in the regulation of cyclin-dependent protein serine/threonine kinase activity, associated with cell cycle regulation. Given the inactivation of serine/threonine kinases ACRE_078410 and ACRE_032470, these mutations may target the inactivation of the same regulatory process. Another nonsense mutation c.15T>G|p.Tyr5* occurred at the beginning of the ACRE_058580 gene and led to the inactivation of the meiotic recombination protein-like protein KFH43389. This protein contains WD40 repeats, which are important for genome integrity and cell cycle progression as they are involved in many processes such as regulation of gene transcription, protein modification, DNA damage and repair, etc. [81]. The considered group of changes also includes mutations at the level of control of replication initiation. Stop loss mutation in the ACRE_032540 (c2137T>A|p.Ter713Lysext*?) may inactivate DNA replication licensing factor RLF, a critical factor required to limit the replication of genomic DNA more than once per cell cycle. This enzyme contains the zinc finger region of primase (Zf-primase) and is the only protein (or part of a complex) that allows the origin of replication to initiate DNA replication at that location in eukaryotes [82,83]. Another frameshift mutation occurred in the ACRE_022590 gene (c.1779dupT|p.Ala594fs), which encodes a regulatory protein that functions at the next stage of the cell cycle, during chromosome segregation during division. This hypothetical protein KFH46929 of 678 AA contains a region for chromosome segregation ATPase Smc, which is responsible for chromosome separation, cell division, and cell cycle control [84,85,86]. However, the disruption of this protein may also be associated with the inactivation of the mutagenesis protection factor (which increased the effectiveness of the effects during CSI), since SMC homolog RecN in response to DNA damage-induced stress contributes to cohesion control [87]. KFH46929 also contains a conserved domain for the mitotic regulator Cut12, which promotes spindle pole activation and integration into the nuclear envelope [88,89]. In addition to these cell cycle-critical changes, a frameshift mutation occurred in the ACRE_009030 gene (c.667delT|p.Tyr223fs) for the cAMP-independent regulatory protein. This transcription factor contains a zf-C4H2 motif and belongs to the Gti1/Pac2 protein family, which has recently been shown to be involved in fungal growth, morphogenesis, stress response, and pathogenicity [90]. The Gti/Pac2 domain is also shared by several important transcription factors involved in dimorphic switching. In *Schizosaccharomyces pombe*, the protein Pac2 controls the onset of sexual development by inhibiting ste11 expression in a pathway that is independent of the cAMP cascade [91]. It is possible that a disruption in this gene led, among other things, to a change in the morphology of *A. chrysogenum* HY, when, during submerged cultivation, the main morphological forms are dumbbell-shaped arthrospores (oidia), whereas in the wild-type strain, mycelial segmentation is not observed (Table 1). The frameshift mutation in ACRE_069720 (c.695delA|p.Lys232fs) disrupts the function of a hypothetical protein with close homology to RhoGAP domain-containing proteins from *Colletotrichum tofieldiae*, *Colletotrichum tofieldiae*, and *Neonectria magnolia*. Proteins with RhoGAP (Rho GTPase-activating *protein domain)* work as molecular switches involved in the regulation of diverse cellular functions including transcription and various cytoskeleton-related events [92,93]. The biosynthesis of secondary metabolites depends on the stages of fungal development, and it is possible that disruption of the factors controlling the cell cycle allows for the intensification of target biosynthesis, going beyond their control [94].

Transcript ACRE_001040 encodes 531 AA putative-like protein with nuclear transport factor 2 (NTF2, 40–161 AA) and RNA recognition motif (RRM, 404–467 AA) domains. This product also contains characteristic motifs for binding to RanGDP repeat-containing nucleoporins and motifs for TAP/p15 interaction (associated with mRNA export through nuclear pore complexes) [95]. The NTF2 domain is involved in the bi-directional transport of macromolecules, ions, and small molecules between the cytoplasm and nucleus. Fusion with the RRM domain and the presence of the TAP/p15-binding motif may indicate involvement in RNA import from the nucleus [96]. This protein has a splicing disorder due to a splice acceptor and intron variant mutation c.1309-2A>C, which may lead to disruption of the functioning of internuclear transport controlled by this protein. The Ran protein (RAs-related Nuclear protein, or GTP-binding nuclear protein Ran) is involved in the processes of RNA and protein translocation through the nuclear pore complex, control of DNA synthesis, cell cycle progression, etc. [97]. Another significant mutation (stop lost p.Ter349Leuext*?) occurred in the ACRE_063590 gene, encoding another putative RNA-binding protein-like protein. This putative product contains two RRM motifs (88–163 AA and 246–324 AA), which may play roles in various processes such as regulating RNA metabolism, stability, and translation within cells [98]. A frameshift mutation in the ACRE_077340 gene (c.1131_1138dupGCTTGGCG|p.Glu380fs) results in an approximately 2-fold truncation of the KFH41538, 678 AA Ran-binding protein (RanBP), with likely loss of some or all activities. RanBP helps maintain a high RanGTP gradient in the nuclear envelope or a low RanGTP level in the cytoplasm. In yeast, RanBP1 (Yrb1) is involved in the release of nuclear export complexes from the cytoplasmic side of the nuclear pore complex and is also capable of shuttling between the nucleus and cytoplasm [99]. The KFH41538 sequence also contains the following: a region for the transcriptional regulator ICP4; the SPRY domain (from SPla and the RYanodine Receptor) involved in many important signaling pathways like RNA processing and regulation of histone H3 methylation; and the C-terminal LisH motif and CTLH domain (CTLH/CRA C-terminal to LisH motif domain), used in various processes such as microtubule dynamics, cell migration, nucleokinesis, and chromosome segregation.

Significant mutations also occurred in proteins associated with epigenetic regulation, particularly chromatin remodeling. A frameshift mutation in the ACRE_068240 gene (c.1058_1064delACTCCTT|p.Asp353fs) resulted in incorrect translation of 955 AA to imitation switch two complex (Isw2) protein-like. This protein contains the WAC_Acf1_DNA_bd (ATP-utilizing chromatin assembly and remodeling) region, as well as the DDT, WHIM1 (WSTF, HB1, Itc1p, and MBD9 motif 1), and WSD (Williams-Beuren syndrome DDT (WSD), D-TOX E motif) domains for histone recognition and DNA binding. In *Saccharomyces cerevisiae*, Isw2, together with another chromatin remodeling factor, Ino80, was shown to regulate checkpoint activity and chromatin structure in the S phase [100]. Deletion of *ISW2* in *S. cerevisiae* extends replicative lifespan [101]. Isw2 is proposed to suppress a cohort of stress response genes, including RAD51; Isw2 inactivation potentiates stress response during aging [100]. In this regard, the inactivation of Isw2 in *A. chrysogenum* HY could also contribute to the activation of stress response elements, since the fungal cell experiences stress with highly active production [102]. Another mutation that could potentially affect the epigenetic status occurred in the ACRE_064570 gene (frameshift c.298delG|p.Glu100fs), encoding a hypothetical protein with the Rxt3 histone deacetylation region, which, as part of the Rpd3L complex, deacetylates lysine residues of the core histones (H2A, H2B, H3, and H4). The mutation may contribute to the alteration of secondary metabolic fluxes in *A. chrysogenum* HY since fungal histone deacetylases can regulate fungal growth, development, and secondary metabolite biosynthesis [103].

#### 2.4.4. Mutations in Other Genes

Significant mutations also arose in genes not involved in biosynthesis, transport or regulation processes (Table 6). For example, such changes occurred in the genes of two ribosomal proteins: in the ACRE_053830 gene (reading frameshift c.359delC|p.Ser120fs), encoding the 40S ribosomal protein S11-B-like protein and in the ACRE_023690 gene (stop lost c.911A>T|p.Ter304Leuext*?), encoding the 60S ribosomal protein L5-like protein. Mutations in ribosomal proteins can have significant effects on the physiological state and other properties of fungi [104,105,106,107,108]. The significance of mutations in S11- and L5-like proteins needs to be clarified. Another mutation that could potentially affect translation is stop lost (c.1531T>C|p.Ter511Glnext*?) in the ACRE_025760 gene, which encodes a tRNA pseudouridine synthase-like protein. This mutation may also be of great importance since it has been shown in model yeast that the loss of individual pseudouridine synthase may affect growth rate, global transcription of amino acid biosynthesis genes, and amino acid levels as well as lipid content [109].

The next two mutations are related to quality control systems. Frameshift variant c.95dupA|p.Ala33fs of the ACRE_034480 gene leads to the inactivation of the 1488 AA machine, UDP-glucose:glycoprotein glucosyltransferase-like protein, which controls the quality of glycosylation. Normally, this endoplasmic reticulum (ER) protein selectively reglycosylates unfolded glycoproteins, thereby ensuring quality control of protein transport from the ER [110]. Disruption of this protein leads to the removal of quality control of glycosylation and the accumulation of errors. A nonsense mutation in the ACRE_049880 gene (c.1162C>T|p.Arg388*) results in dysfunction of the mitochondrial chaperone protein CLPX-like of 595 AA. This caseinolytic mitochondrial matrix peptidase is involved in protein quality control through ATP-dependent proteolysis leading to protein catabolism [111]. This protein functions as part of the mitochondrial CLPXP complex, which, like bacterial CLPXP, consists of a core 14-mer proteolytic chamber CLPP (assembled from two 7-mer CLPP rings) and one or two 6-mer CLPX chaperone caps (type AAA+) that recognize and unfold substrate proteins [112]. It is also suggested that in fungi, the function of mitochondrial CLPXP is in the control and/or maintenance of energy metabolism [111]. It was also shown that deletion of the *PaClpP* gene (encoding the proteolytic subunit of CLPXP in *Podospora anserina*) leads to an unexpected healthy lifespan phenotype in the mutant strain [113]. In this regard, the mutation in the ACRE_049880 gene in *A. chrysogenum* HY could both lead to a redistribution of energy flows in the cell and lead to the formation of a healthy lifespan phenotype.

The disruption in the ACRE_029210 gene (Stop lost c.449A>G|p.Ter150Trpext*?), encoding a calmodulin-like protein, may have a pleiotropic effect, since calmodulin can be involved in a wide variety of processes, such as regulating stress responses, morphogenesis, pathogenesis, and regulation of secondary metabolism [114,115]. Another significant mutation that may be associated with a change in the physiology of *A. chrysogenum* HY occurred at the beginning of the ACRE_02341 gene (frameshift c.41delC|p.Pro14fs) and leads to a protein that has a region characteristic of spherulation-specific family 4. This protein is associated with the process of spherulation, the formation of spores, under starvation conditions. During CSI, *A. chrysogenum* HY lost the ability to form conidia, which may be associated with the loss of spherulin function [116]. A frameshift mutation in the ACRE_031710 gene (c.616_620delAGGGG|p.Arg206fs) disrupts the function of the hypothetical protein with CAP (cysteine-rich secretory proteins, antigen 5, and pathogenesis-related 1 protein) domain. The role of this mutation is difficult to assess due to the complex effects of proteins belonging to the CAP superfamily [117,118].

Significant mutations occurred in several other transcripts whose role is not yet understood, such as a frameshift in the ACRE_036450 gene (c.409delC|p.Leu137fs), or a nonsense mutation in the ACRE_009970 gene (c.601C>T|p.Gln201*), or a loss of a stop in the ACRE_063290 gene (c.562T>C|p.Ter188Glnext*?).

## 3. Discussion

In order to obtain high-yielding CPC production in *A. chrysogenum* strains, it is not enough to simply optimize the cultivation conditions of natural isolates (or their analogs in the early stages of CSI programs); it is necessary to introduce complex and global changes at the genetic level. Thus, in a recent study, optimization of fermentation conditions in laboratory fermentor (14 L) for *A. chrysogenum* strain W42-I (an intermediate strain from the W42 strain improvement program) resulted in a maximum CPC yield of 0.399 g/L [119]. The *A. chrysogenum* HY (RNCM 408D) strain studied in this work produces 12 g/L CPC and more. At the genetic level, this is expressed in the 3472 mutations detected. This rather significant number of changes is consistent with the number of SNPs identified in the *Penicillium chrysogenum* strain DS17690, an improved penicillin G (PenG) producer (Table 7) [41]. Strain DS17690 (PenG yield—95 mg/gDW) was obtained in the second period of the CSI based on Wisconsin 54-1244 (PenG yield—20 mg/gDW), which in turn was obtained in the first period of the CSI based on the natural isolate *P. chrysogenum* NRRL 1951 (PenG yield—4 mg/gDW). Strain Wisconsin 54-1244 carries 455 SNPs relative to NRRL 1951; strain DS17690 carries 2056 SNPs relative to Wisconsin 54-1244, and 2511 SNPs relative to the original isolate NRRL 1951 (Table 7).

A characteristic feature is that the number of mutations in the HIGH category (termination mutations, frameshift, and nonsense mutations) is less than in other categories. However, the number of mutations from the MODERATE category (non-synonymous) is greater than the number of mutations from the LOW category (synonymous). Thus, in *A. chrysogenum* HY (RNCM 408D), 532 non-synonymous and 373 synonymous variants were detected (Table 4). The Wisconsin 54-1244 strain has 151 non-synonymous and 55 synonymous substitutions compared to natural isolate NRRL 1951; the number of mutations in the DS17690 strain increases significantly and amounts to 709 non-synonymous and 326 synonymous substitutions related to NRRL 1951. The same ratio between the number of mutations in the HIGH, MODERATE, and LOW categories is observed for other improved fungal strains [42].

In *A. chrysogenum* HY, mutational changes arising from the CSI program were found in 34 of the 48 BGCs examined. For reasons that are not entirely clear, clusters with PKS turned out to be the most preferred target. All PKS in *A. chrysogenum* belong to type I, iterative, which is typical for fungal PKS [122]. A total of 16 mutations were found in 11 of 21 PKS sequences, 9 of which (in 8 PKS) belong to the MODERATE category (and carry missense mutations), and one, in PKS 10, belongs to the HIGH category (stop gained p.Tyr699*) and leads to the appearance of a stop codon after 699 AA in the sequence encoding 2566 AA. The total size of PKS sequences is 163,452 bp, which is 0.57% of the size of the *A. chrysogenum* WT genome, estimated at 28,600,000 bp [53]. In *A. chrysogenum* HY, 961 mutations affecting coding sequences were detected (56—HIGH, 532—MODERATE, and 373—LOW); their frequency is 3.36 × 10^−5^. At the same time, the mutation frequency in PKS sequences is higher, more than 3 times, and amounts to 9.79 × 10^−5^ (16 mutations among 163,452 bp). This may indicate that PKS sequences were among the preferred targets for random mutagenesis in the *A. chrysogenum* HY improvement program. These data are consistent with the changes observed in the improved penG producer, *P. chrysogenum* DS68530; among the backbone enzymes, PKS was the most favored target for mutations [41]. It is possible that the inactivation of secondary metabolisms associated with the consumption of acetyl-CoA and manoyl-CoA is necessary for the highly active production of beta-lactams. In any case, both highly active beta-lactam producers, *P. chrysogenum* DS68530 and *A. chrysogenum* HY, have inactivated the metabolism of pigment polyketides of sorbicillinoids.

Sorbicillinoids are synthesized after the transition of filamentous fungi such as *P. chrysogenum* or *A. chrysogenum* from the trophophase to the idiophase and are among the most undesirable by-products of industrial fermentation of target secondary metabolite. This is due to the fact that such pigment compounds are synthesized in a planned manner under the influence of internal signals, unlike many other secondary metabolites that can be synthesized if the appropriate exotic signals arise, which does not interfere with the target production at optimized fermentation stages. Therefore, sorbicillinoids will be synthesized under virtually any fermentation conditions and draw off resources that could potentially be used to synthesize the target secondary metabolite. Therefore, in CSI programs, industrial producers often bleach, i.e., become uncolored, since they lose the ability to synthesize undesirable pigment metabolites [62].

As shown previously, improved fungal strains can be characterized not only by a decrease in pigment production but also by virulence [102]. The putative Nudix hydrolase, which may be a phytopathogenic factor [71], is inactivated in *A. chrysogenum* HY as a result of a frameshift mutation at the beginning of the ACRE_007400 gene. A similar mutation occurred in the ACRE_009030 gene, leading to the inactivation of the encoded putative cAMP-independent regulatory protein containing the Gti1_Pac2 domain, which has recently been shown to be involved in pathogenicity [90].

*A. chrysogenum* HY also appears to have impaired nucleo-cytoplasm transport of macromolecules, due to defects in nuclear transport factor 2 and Ran-binding protein-like protein. A mutation was found in ACRE_064050 dicer-like protein-like protein, ribonuclease III-like enzyme playing a key role in the RNA silencing pathway [123] through regulation of the production of small RNAs in eukaryotes [124]. A series of mutations unexpectedly affected the cell cycle during the S phase (replication license, Isw2 checkpoint activity, and chromatin structure in the S phase) and the M phase. The role of mutation in chromatin remodeling factor Isw2 can be considered from a different perspective, focusing on the state of chromatin at loci containing BGCs for target metabolism. ISW has been shown to be required for transcriptional repression, nucleosome organization, and establishment of typical histone methylation patterns in facultative heterochromatin domains [125,126]. On the other hand, a paradigm has emerged that postulates that chromatin remodeling, associated with the biosynthesis of secondary metabolites in fungi, is the responsibility of several enzymes capable of introducing individual modifications into histones, such as methylation and acetylation and other modifications as follows: (i) histone methyltransferase LaeA, which works in combination with regulatory proteins of the velvet complex [127,128,129]; histone acetyltransferase GcnE [130]; and histone deacetylase SirE [131]. The greatest efforts are concentrated on the study of LaeA and velvet complex proteins [132,133,134,135,136]. Changes in the LaeA/velvet system were shown to be key events that occurred during the creation of the studied improved fungal producers in the CSI programs [41,137]. However, there are no comparisons in the literature where changes in the chromatin remodeling system (and its ISW component) can also affect secondary metabolism, for example, as a result of remodeling in the corresponding BGC locus from heterochromatin to euchromatin.

A series of mutations associated with a decrease in SAMe consumption in *A. chrysogenum* HY may intensify the metabolism of sulfur required to build beta-lactam molecules, where cysteine is used as a precursor, but the most important stimulator of industrial production is methionine. Methionine has been shown to stimulate CPC production by enhancing the accumulation of endogenous SAMe [138]. It is possible that this stimulation with an increase in the SAMe pool is realized, among other things, as a result of the work of LaeA, S-adenosyl-L-methionine-dependent histone methylase and a positive globulin regulator of beta-lactams [127]. On the other hand, pool SAMe is used to obtain a decarboxylated analog, which then acts as a donor in the aminopropylation reaction in the biosynthesis of polyamines (spermidine from putrescine and spermine from spermidine) [139]. The demonstrated 4–5-fold increase in polyamine content in *A. chrysogenum* HY [31] may also affect the SAMe pool. It has been previously discussed that the polyamine content of this strain could have arisen as a result of side mutational events in the CSI program, allowing the cell to survive sublethal mutagenic effects as a result of protection from reactive oxygen species and participation in reparation processes [140,141]. It is possible that the increase in polyamine content also affects the SAMe pool. In any case, exogenous administration of polyamines leads to an additional increase in cephalosporin C production, which may be associated with the inactivation of endogenous polyamine biosynthesis (by a feedback mechanism) [142] and a decrease in the load on the SAMe pool. The key enzyme for the biosynthesis and regulation of polyamines is ODC; the resistance of *A. chrysogenum* HY to ODC inhibitors is described in more detail in the Section 1. In this regard, the missense mutation in the ACRE_055510 gene (c.962A>T|p.Tyr321Phe), encoding ornithine decarboxylase-like protein, may play a significant role in the activity of this enzyme (Table S1).

The identified mutations in the genome of *A. chrysogenum* HY, a highly active producer of cephalosporin C, open up the possibility for further targeted studies to establish the role of these changes. For example, after identifying changes in the genome of *P. chrysogenum* DS17690 [41], the authors, in a series of elegant experiments, elucidated the role of two missense mutations in the polyketide synthase genes *sorA* and *sorB* in the sorbicillinoid BGC [62].

The mutations revealed belong to a variety of groups, but several types of mutations can be distinguished that affect processes that have previously been identified in multi-omics experiments with improved fungal producers. First, they occurred in 34 of the 47 BGCs studied in alternative gene clusters, some of which are inactivated due to significant mutations in central genes. Such events were observed for the classically improved producer *P. chrysogenum* DS17690; the largest number of mutations were also found for PKS [41]. Among the alternative BGCs, *A. chrysogenum* HY has a disrupted PKS in the sorbicillinoid pigment cluster; disruptions in this cluster lead to bleaching of industrial fungal strains, which is important since the cell does not spend resources on pigment synthesis [62]. In addition, mutations affect the chromatin remodeling system, which plays a key role, including in the regulation of secondary metabolite biosynthesis in fungi [137,143], and can be disrupted in highly active fungal producers [41]. Also, mutations affecting primary metabolism were identified in *A. chrysogenum* HY, which was shown in a previously published work with an improved producer *Penicillium rubens* [144]. In addition to the series of previously described mutations for improved producers, a group of changes associated with changes in sulfur and SAMe metabolism, disturbances in the cell quality control systems, the life cycle and transport of macromolecules between the nucleus and cytoplasm, and the transport of metabolites from the cell and between its compartments have been characterized in *A. chrysogenum* HY. It is possible that some of these changes are also universal since they are necessary in the process of converting a WT fungal strain into a biofactory for the production of a target secondary metabolite.

The identified genomic changes may correlate with the biosynthetic pathway and regulatory mechanisms involved in the production of cephalosporin C beta-lactams [18,57,145], but specific conclusions can be made after conducting targeted experiments introducing similar mutations into the wild-type strain and complementing the mutations under study.

## 4. Materials and Methods

### 4.1. Strains of Microorganisms

Strain *A. chrysogenum* RNCM 408D (HY, high-yielding CPC producer, [22]) was used for genomic DNA extraction; GenBank accession number: JBIHMN010000000 (this study). The genomic DNA sequence of *A. chrysogenum* ATCC 11550 (WT, wild type isolate Brotzu, [146]; GenBank accession number: JPKY00000000.1, [53]) was used as a reference to evaluate the changes that occurred in the genome of *A. chrysogenum* RNCM 408D.

### 4.2. Isolation of gDNA from A. chrysogenum HY

*A. chrysogenum* HY was pre-cultured on slanted agarized CPA medium (40 g/L maltose, 10 g/L peptone, 20 g/L malt extract, 25 g/L agar, pH 7.0–7.4) for 12 days. Then the fungal cells were collected with 5 mL of Czapek-Dox medium (30 g/L sucrose, 2 g/L NaNO_3_, 1 g/L K_2_HPO_4_, 0.5 g/L MgSO_4_ × 7H_2_O, 0.5 g/L KCl, 0.01 g/L FeSO_4_ × 7 H_2_O, 25 g/L agar, pH 7.0–7.4), transferred to a 250 mL Erlenmeyer flask with 20 mL of Czapek-Dox medium, incubated for 48 h, 28 °C on a CERTOMAT^®^BS-1 shaker (“Sartorius”, Göttingen, Germany) at 230 rpm. The resulting biomass was washed three times with 5–20 volumes of H_2_O, 300 µL and placed in Eppendorf’s, 1.5 mL, lyophilized, and resuspended in 200 µL of TES buffer (1% SDS, 1 mM EDTA, 100 mM Tris-HCl, pH 8.5). The mixture was shaken vigorously on a Vortex for 3 min, incubated at 65 °C for 1 h, and periodically shaken on a Vortex for 1 min. The mixture was cooled to room temperature, 200 µL of phenol (saturated with 0.2 M Tris-HCl, pH 8.5) and 0.5 volume of glass beads (D = 500 µm) were added, shaken on a Vortex for 3 min, and centrifuged for 5 min at 13,400 rpm on a MiniSpin apparatus (Eppendorf, Hamburg, Germany). The upper aqueous fraction was then collected, washed with an equal volume of a chloroform/isoamyl alcohol mixture (24:1), shaken for 1 min, incubated for 5 min at room temperature, shaken again for 1 min, and centrifuged for 5 min at 13,400 rpm. The upper aqueous fraction was collected, 1/10 volume of 3M potassium acetate (pH 5.2), and 3 volumes of 96% chilled ethyl alcohol were added. Incubated for 20 min, −20 °C, centrifuged at 13,400 rpm, the sediment was washed with cooled 70% ethyl alcohol, dried, and resuspended in 20 µL H_2_O.

### 4.3. Whole Genome Sequencing and Assembly

The isolated gDNA was fragmented by ultrasound into fragments with an average length of 400 nucleotides using a Covaris S2 device (Covaris, Woburn, MA, USA) in accordance with the manufacturer’s recommendations. The resulting material was used to prepare a library with the NEBNext^®^ Ultra™ II DNA Library Prep Kit for Illumina (New England Biolabs, Ipswich, MA, USA). Final DNA concentrations were measured using the Qubit Quant-iT dsDNA HS Assay kit (Thermo Fisher Scientific, Waltham, MA, USA). The created library was used for sequencing on a HiSeq1500 device (Illumina, San Diego, CA, USA) in single-end read mode, 250 nucleotides long. Sequencing resulted in 61,204,569 reads (GenBank: SRR30996808). These raw data were used for de novo assembly using the SPAdes Genome Assembler v. 3.9.0 program (Illumina, San Diego, CA, USA). The determined sequences (GenBank: PRJNA1168918) were compared with previously annotated sequences for the genome of the wild-type strain *A. chrysogenum* ATCC 11550 (GenBank: JPKY00000000.1). Data on insertions, deletions, and single nucleotide polymorphisms were obtained using the GATK v. 4.6.0.0 program [147], available online [148].

### 4.4. Defining BGCs and Performing Comparative Analysis

BGCs in the *A. chrysogenum* genome sequences were determined using antiSMASH fungal version 7.0 software, available online [54,149]. The following additional software modules, integrated into antiSMASH, were also used: MIBiG cluster comparison, Pfam cluster analysis, Pfam-based GO term annotation, SubClusterBlast analysis, RREFinder, and TIGRFam analysis. BGCs were also detected using the Cluster Boundary Prediction from Transcription Factor Binding Sites (CASSIS) mode [54,149]. To more fully unlock the biosynthetic potential of *A. chrysogenum*, the detection stringency parameters ranged from strict (detects well-defined clusters containing all required moieties) to relaxed (detects partial clusters missing one or more functional moieties) and loose (detects poorly defined clusters and clusters likely to correspond to primary metabolites). Sequence comparisons were performed using BLAST+ v. 2.16.0, available online [150]. Sequence alignment was performed in Vector NTI v. 11.5 (Thermo Fisher Scientific Inc., Waltham, MA, USA) [151].

## 5. Conclusions

This work reveals mutational changes that arose in the genome of a highly active cephalosporin C producer, the *A. chrysogenum* RNCM 408D strain (HY, high-yielding CPC producer) during the classical strain improvement program (CSI). This strain has been intensively studied over the past 20 years, as a result of which changes in its physiology, morphology, and karyotype have been characterized, and numerous studies have been conducted that relate to the molecular basis for the increase in cephalosporin C production; however, mutational changes in the genome have not been comprehensively characterized. In this work, a total of 3472 changes were identified, 56 of which resulted in significant changes in the protein product, such as (i) enzymes for the biosynthesis of primary and secondary metabolites; (ii) transporters, including MDR; (iii) regulators of various processes, ranging from cell cycle control to chromatin remodeling factors; and (iv) other proteins. Particular attention was paid to the mutations that arose in biosynthetic gene clusters (BGCs). Surprisingly, there were no mutations in the “early and late” BGCs of beta-lactams, except for one regulatory mutation in the downstream region of *cefG*. It is possible that trans-acting regulatory factors, including those of an epigenetic nature, contribute to the 5 to 300-fold upregulation of these genes. Among the alternative BGCs, multiple mutations were found, which affected, among other things, the spine genes; mutations are especially common in the polyketide synthase genes.

The obtained data allow us not only to establish the molecular basis that led to an increase in the production of cephalosporin C in *A. chrysogenum* HY but also to identify certain universal changes that occur in fungal genomes during the creation of a highly active producer of secondary metabolites using classical methods.

## Figures and Tables

**Table 1:** Main phenotypic differences between *A. chrysogenum* WT and HY strains.

Nutrient Medium	Phenotype	*A. chrysogenum* WT	*A. chrysogenum* HY	Reference
Complex agarized (CPA) medium	Colony size, mm ^1^	12–15	0.5–5	[26]
	Heterogeneity in colony size	−	+	
	Yellow-cream coloration	+	−	
	Aerial mycelium	+	−	[28]
Czapek-Dox agarized (CDA) medium	Conidia formation	Normal	Reduced	
	Sensitivity to ODC inhibitors	High	Low	[33]
Czapek-Dox (CD) medium	Intracellular polyamine content	Normal	Increased by 4–5 times	
Complex (CP) medium	Cell wall	Normal	Thinned	[29]
	Intracellular ATP content	Normal	Reduced by 3–4 times	[30,31]
	Activity of plasma membrane H^+^-ATPase	Normal	Reduced by ~2 times	
	Main morphological forms during CPC production	Mycelium, conidia	Oidia (or arthrospores)	[32]
	Dry biomass	Normal	Reduced by ~2 times	[27]
	**CPC production, mg/L**	**25–70**	**12,000+**	

^1^ After cultivation for 12 days at 28 °C.

**Table 2:** Number of SNPs within different categories found in the genome of *A. chrysogenum* HY strain compared to *A. chrysogenum* WT.

Category of Mutation	Number of Mutations
HIGH	56
MODERATE	532
LOW	373
MODIFIER	2769
**TOTAL _by categories_**	**3730**
**TOTAL _in genome_**	**3472** ^1^

^1^ The number of mutations by category (3730) is greater than the total number of mutations in the genome (3472) since a single mutation can belong to several categories.

**Table 3:** Mutations in biosynthetic genes of *A. chrysogenum* HY related to the HIGH category.

Gene	Product			Mutation	
	*Accession №*	*Length, AA*	*Annotated as*	*Type*	*Position*
ACRE_072830	KFH41986	500	Aldehyde dehydrogenase-like protein	Stop gained	c.452C>A|p.Ser151*
ACRE_051330	KFH44073	569	Succinyl-CoA--L-malate CoA-transferase beta subunit-like	Stop gained	c.1355C>A|p.Ser452*
ACRE_025660	KFH46670	581	Putative epoxide hydrolase-like	Frameshift variant	c.454delC| p.Leu152fs
ACRE_061140	KFH43164	557	Pisatin demethylase-like protein	Frameshift variant	c.980delA|p.Tyr327fs
ACRE_071740	KFH42113	298	Formyltetrahydrofolate deformylase-like protein	Stop lost and splice region variant	c.896A>C| p.Ter299Serext*?
ACRE_067920	KFH42477	475	Esterase-like protein	Stop gained	c.979C>T|p.Arg327*
ACRE_009560	KFH48096	306	Peroxisomal 2,4-dienoyl-CoA reductase (NADPH2)	Frameshift variant	c.44dupA|p.Asp15fs
ACRE_090470	KFH40296	419	Putative CDP-alcohol phosphatidyltransferase class-I family protein-like	Stop gained	c.26T>G|p.Leu9*
ACRE_054620	KFH43751	627	Phospholipid:diacylglycerol acyltransferase-like protein	Stop gained	c.878T>A|p.Leu293*
ACRE_084300	KFH40859	560	Hypothetical protein (with galactosyltransferase domain)	Stop gained	c.1093G>T|p.Glu365*
ACRE_070590	KFH42213	236	NAD(P)H-hydrate epimerase-like protein	Stop gained and splice region variant	c.280C>T|p.Gln94*
ACRE_007400	KFH48403	207	Hypothetical protein (with NUDIX_Hydrolase region)	Frameshift variant and start lost	c.1dupA|p.Met1fs
ACRE_081590	KFH41138	355	4-dimethylallyltryptophan N-methyltransferase-like protein	Stop gained	c.805C>T|p.Arg269*
ACRE_065750	KFH42725	398	1-aminocyclopropane-1-carboxylate synthase-like protein	Frameshift variant	c.1107_1110dupCCCG| p.Lys371fs
ACRE_012290	KFH47892	8601	Nonribosomal peptide synthetases 4	Stop gained	c.19633C>T|p.Gln6545*
ACRE_060650	KFH43187	2566	Polyketide synthase 10	Stop gained	c.2097T>A|p.Tyr699*

**Table 4:** Mutations in transporter genes of *A. chrysogenum* HY related to the HIGH category.

Gene	Product				Mutation	
	*Accession №*	*Length, AA*	*Annotated as*	*Transporter Class*	*Type*	*Position*
ACRE_087850	KFH40525	1142	Calcium-transporting ATPase-like	P-type Na^+^-ATPase	Stop gained	1577G>A|p.Trp526*
ACRE_075010	KFH41796	1549	Metal resistance protein-like	ABC	Splice donor variant and intron variant	c.3159+1G>A
ACRE_057310	KFH43509	487	Transporter-like protein	MFS	Frameshift variant	c.416_420delTGGGA|p.Val139fs
ACRE_072140	KFH42066	433	D-galactonate transporter-like	MFS	Stop lost and splice region	1300T>C|p.Ter434Glnext*?
ACRE_045230	KFH4469	538	Hypothetical protein (with DinF/NorM/MATE region for Na^+^-driven multidrug efflux pump)	MATE	Splice acceptor variant and intron variant	c.1054-2A>G

**Table 5:** Mutations in regulatory genes of *A. chrysogenum* HY related to the HIGH category.

Gene	Product				Mutation	
	*Accession №*	*Length, AA*	*Annotated as*	*Regulatory Motive*	*Type*	*Position*
ACRE_051010	KFH44144	290	Hypothetical protein (putative bZIP transcription factor)	bZIP	Frameshift variant	c.483dupA|p.Val162fs
ACRE_021680	KFH47037	399	Hypothetical protein (homologous to fungal transcription activators)	-	Stop gained	c.373A>T|p.Lys125*
ACRE_072410	KFH42038	1116	Hypothetical protein (with Zf C3H1 motif)	ICP4 Zf C3H1	Stop gained	c.1910C>A|p.Ser637*
ACRE_088010	KFH40507	783	R3H domain-containing protein-like	ICP4, R3H, SUZ, FtsK	Stop gained	c.55G>T|p.Glu19*
ACRE_043640	KFH44855	413	Serine/threonine-protein kinase-like	Ser/Thr PK	Stop gained and splice region variant	c.1148T>A|p.Leu383*
ACRE_078410	KFH41460	437	Protein kinase-like protein	Ser/Thr PK	Frameshift variant	c.450delC|p.Ile151fs
ACRE_032470	KFH45953	986	Protein kinase-like protein	PK_SCY1	Stop gained	c.2401C>T|p.Gln801*
ACRE_045800	KFH44600	1216	Stress response-like protein	NST1 BARX RCC4	Frameshift variant	c.2228delA|p.Lys743fs
ACRE_084130	KFH40881	333	Meiotically up-regulated gene 80 protein-like protein	CYCLINScPCL1	Stop gained	c.777G>A|p.Trp259*
ACRE_058580	KFH43389	318	Meiotic recombination protein-like protein	WD40	Stop gained	c.15T>G|p.Tyr5*
ACRE_032540	KFH45961	712	DNA replication licensing factor-like protein	Zf-primase	Stop lost and splice region variant	c.2137T>A|p.Ter713Lysext*?
ACRE_022590	KFH46929	678	Hypothetical protein(with Smc domain)	Smc Cut12	Frameshift variant	c.1779dupT|p.Ala594fs
ACRE_009030	KFH48345	439	cAMP-independent regulatory protein-like protein	Gti1_Pac2 zf-C4H2	Frameshift variant	c.667delT|p.Tyr223fs
ACRE_069720	KFH42294	248	Hypothetical protein (close to rhoGAP proteins)	rhoGAP	Frameshift variant	c.695delA|p.Lys232fs
ACRE_001040	KFH48908	531	Putative-like protein (with NTF2, RRM)	NTF2 RRM	Splice acceptor variant and intron variant	c.1309-2A>C
ACRE_063590	KFH42915	348	Putative RNA-binding protein-like protein	RRM	Stop lost and splice region variant	p.Ter349Leuext*?
ACRE_077340	KFH41538	687	Ran-binding protein-like	Ran-BPM ICP4 SPRY-LisH	Frameshift variant	c.1131_1138dupGCTTGGCG|p.Glu380fs
ACRE_068240	KFH42443	955	Imitation switch two complex protein-like protein	WAC_Acf1_DNA_bd DDT WHIM1 WSD	Frameshift variant	c.1058_1064delACTCCTT|p.Asp353fs
ACRE_064570	KFH42797	1081	Hypothetical protein (with region for histone deacetylation protein Rxt3)	CAF1	Frameshift variant	c.298delG|p.Glu100fs

**Table 6:** Mutations in other genes of *A. chrysogenum* HY related to the HIGH category.

Gene	Product			Mutation	
	*Accession №*	*Length, AA*	*Annotated as*	*Type*	*Position*
ACRE_053830	KFH43833	225	40S ribosomal protein S11-B-like protein	Frameshift variant	c.359delC|p.Ser120fs
ACRE_023690	KFH46815	303	60S ribosomal protein L5-like protein	Stop lost and splice region variant	c.911A>T|p.Ter304Leuext*?
ACRE_025760	KFH46655	510	tRNA pseudouridine synthase-like protein	Stop lost and splice region variant	c.1531T>C|p.Ter511Glnext*?
ACRE_034480	KFH45737	1488	UDP-glucose:glycoprotein glucosyltransferase-like protein	Frameshift variant	c.95dupA|p.Ala33fs
ACRE_049880	KFH44226	595	Mitochondrial clpX-like chaperone-like protein	Stop gained	c.1162C>T|p.Arg388*
ACRE_029210	KFH46306	149	Calmodulin-like protein	Stop lost and splice region variant	c.449A>G|p.Ter150Trpext*?
ACRE_02341	KFH46844	225	Spherulin-like protein	Frameshift variant	c.41delC|p.Pro14fs
ACRE_031710	KFH46018	248	Hypothetical protein (with CAP domain)	Frameshift variant	c.616_620delAGGGG|p.Arg206fs

**Table 7:** Mutational impact of CSI on a *P. chrysogenum* (NRRL 1951 → Wisconsin 54-1244 → DS17690) and *A. chrysogenum* (ATCC 11550 → RNCM 408D) strain lineages.

Strain	Total	Non-Cording	Cording					Reference
			Synonymous	Non-Synonymous	Termination Mutation	Frameshift	Nonsense	
Wis54-1244 ^1^	455	240	55	151	2	6	1	[120]
DS176902nd _SCI_ ^2^	2056	1187	271	558	3	13	24	[121]
DS17690_Total_ ^3^	2511	1427	326	709	5	19	25	[41]
RNCM 408D ^4^	3472	2511	373	532	12	23	21	current

^1^ relative to *P. chrysogenum* NRRL1951; ^2^ relative to *P. chrysogenum* Wisconsin 54-1244; ^3^ relative to *P. chrysogenum* NRRL1951; ^4^ relative to *P. chrysogenum A. chrysogenum* WT (ATCC 11550).

## Data Availability

The data are contained within the article.

## Supplementary Materials

The supporting information can be downloaded at: https://www.mdpi.com/article/10.3390/ijms26010181/s1.
