# Supplementary material for: Comparative Genomic Analysis Reveals Key Changes in the Genome of Acremonium chrysogenum That Occurred During Classical Strain Improvement for Production of Antibiotic Cephalosporin C
Source: Int J Mol Sci. 2024 Dec 28;26(1):181. doi: 10.3390/ijms26010181 (PMC11719821; doi:10.3390/ijms26010181)
Supplement: Supplementary file 1 [file ijms-26-00181-s001.zip › Table S2.pdf]

International Journal of *Molecular Sciences*

MDPI

Table S2. Types of mutations within categories found in the genome of *A. chrysogenum* HY strain compared to *A. chrysogenum* WT.

Category of mutation	Type of mutation	Number of mutations
HIGH	Frameshift variant	22
	Frameshift variant & start lost	1
	Stop gained	19
	Stop gained & splice region variant	2
	Stop lost	9
	Splice acceptor variant & intron variant	2
	Splice donor variant & intron variant	1
MODERATE	Missense variant	527
	Disruptive inframe insertion	1
	Conservative inframe insertion	1
	Conservative inframe deletion	2
	Disruptive inframe deletion	1
LOW	Synonymous variant	355
	Splice region variant & synonymous variant	4
	Splice region variant & intron variant	14
MODIFIER	Intergenic region	2323
	Upstream gene variant	878
	Downstream gene variant	863
	Intron variant	176
