# Supplementary material for: Comparative Genomic Analysis Reveals Key Changes in the Genome of Acremonium chrysogenum That Occurred During Classical Strain Improvement for Production of Antibiotic Cephalosporin C
Source: Int J Mol Sci. 2024 Dec 28;26(1):181. doi: 10.3390/ijms26010181 (PMC11719821; doi:10.3390/ijms26010181)
Supplement: Supplementary file 1 [file ijms-26-00181-s001.zip › Table S3.pdf]

Table S3. Biosynthetic gene clusters in the *A. chrysogenum* WT genome.

Tool	NRPS	PKS	TPC	Hybrid	Other	Total	GenBank №, referense
antiSMASH v. *2.0*	7	14	10	8	3	42	JPKY00000000.1, [1]
antiSMASH v. *7.0*	9	14	9	9	9	50	JPKY00000000.1, current

1. Terfehr, D.; Dahlmann, T.A.; Specht, T.; Zadra, I.; Kürnsteiner, H.; Kück, U. Genome Sequence and Annotation of Acremonium chrysogenum, Producer of the β-Lactam Antibiotic Cephalosporin C. *Genome Announc.* **2014**, *2*, e00948-14-e00948-14, doi:10.1128/genomeA.00948-14.
