# Supplementary material for: Comparative Genomic Analysis Reveals Key Changes in the Genome of Acremonium chrysogenum That Occurred During Classical Strain Improvement for Production of Antibiotic Cephalosporin C
Source: Int J Mol Sci. 2024 Dec 28;26(1):181. doi: 10.3390/ijms26010181 (PMC11719821; doi:10.3390/ijms26010181)
Supplement: Supplementary file 1 [file ijms-26-00181-s001.zip › Tables caption.pdf]

Table S1: Mutations arising in the *A. chrysogenum* RNCM 408D (HY) strain after the CSI program relative to the initial *A. chrysogenum* ATCC 11550 (WT) strain

Table S2: Types of mutations within categories found in the genome of *A. chrysogenum* HY strain compared to *A. chrysogenum* WT

Table S3: Biosynthetic gene clusters in the *A. chrysogenum* WT genome

Table S4: Mutations in BGCs of *A. chrysogenum* HY;

Table S5: Mutations related to the HIGH category in *A. chrysogenum* HY.
